# Uncoupled turnover disrupts mitochondrial quality control in diabetic retinopathy

**DOI:** 10.1172/jci.insight.129760

**Published:** 2019-12-05

**Authors:** Jose R. Hombrebueno, Lauren Cairns, Louise R. Dutton, Timothy J. Lyons, Derek P. Brazil, Paul Moynagh, Tim M. Curtis, Heping Xu

**Affiliations:** 1 Wellcome-Wolfson Institute for Experimental Medicine, School of Medicine, Dentistry and Biomedical Sciences, Queen’s University Belfast, Belfast, United Kingdom.; 2 Institute of Inflammation and Ageing, College of Medical and Dental Sciences, University of Birmingham, Birmingham, United Kingdom .; 3 Division of Endocrinology and Diabetes, Medical University of South Carolina, Charleston, South Carolina, USA.; 4 Institute of Immunology, Department of Biology, National University of Ireland Maynooth, Maynooth, County Kildare, Ireland.

**Keywords:** Neuroscience, Ophthalmology, Autophagy, Mitochondria, Retinopathy

## Abstract

Mitochondrial quality control (MQC) is crucial for regulating CNS homeostasis, and its disruption has been implicated in the pathogenesis of some of the most common neurodegenerative diseases. In healthy tissues, the maintenance of MQC depends upon an exquisite balance between mitophagy (removal of damaged mitochondria by autophagy) and biogenesis (de novo synthesis of mitochondria). Here, we show that mitophagy is disrupted in diabetic retinopathy (DR) and decoupled from mitochondrial biogenesis during the progression of the disease. Diabetic retinas from human postmortem donors and experimental mice exhibit a net loss of mitochondrial contents during the early stages of the disease process. Using diabetic mitophagy-reporter mice (*mitoQC*-*Ins2^Akita^*) alongside *pMitoTimer* (a molecular clock to address mitochondrial age dynamics), we demonstrate that mitochondrial loss arose due to an inability of mitochondrial biogenesis to compensate for diabetes-exacerbated mitophagy. However, as diabetes duration increases, Pink1-dependent mitophagy deteriorates, leading to the build-up of mitochondria primed for degradation in DR. Impairment of mitophagy during prolonged diabetes is linked with the development of retinal senescence, a phenotype that blunted hyperglycemia-induced mitophagy in *mitoQC* primary Müller cells. Our findings suggest that normalizing mitochondrial turnover may preserve MQC and provide therapeutic options for the management of DR-associated complications.

## Abstract 2

Uncoupled mitophagy and mitochondrial biogenesis leads to mitochondrial damage in the retina during the progression of diabetes.

## Graphical abstract

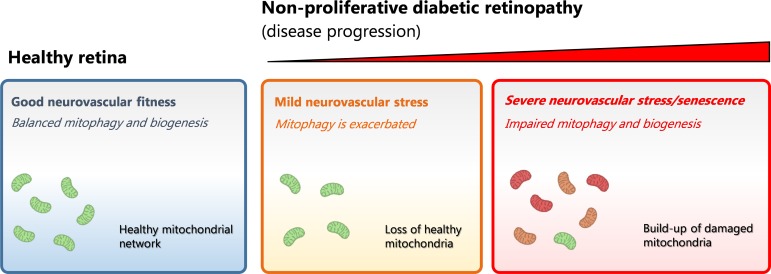

## Introduction

Diabetic retinopathy (DR) is a leading cause of blindness in the working-age population (1, 2). It is characterized by a progressive dysfunction of the retinal neurons, glial cells, and microvasculature, leading to abnormal vessel proliferation and vascular leakage that threaten vision (3). Although the pathological hallmarks of DR are well defined, how sustained hyperglycemia leads to retinal neurovascular dysfunction remains to be elucidated. The pathogenesis of DR is complex and driven by a multitude of factors in addition to hyperglycemia, including oxidative stress, dyslipidaemia, and chronic parainflammation (4). Current therapies for DR remain unsatisfactory and focus mainly on targeting the end-stages of the disease process (5). Consequently, there is an urgent need to develop new interventions — particularly those that are able to prevent the initiation and development of this condition.

A growing body of evidence suggests that mitochondrial dysfunction plays a pivotal role in the early pathogenesis of DR. For example, mitochondrial DNA (mtDNA) damage, mitochondrial overproduction of ROS, and inefficient mtDNA repair mechanisms have been implicated both in the human diabetic retina and animal models of DR (6). At the ultrastructural level, mitochondrial damage in retinal endothelial cells and neurons during DR is evidenced by the presence of vacuolated mitochondria with disruption of the lamellar cristae (7). Mitochondrial changes have also been observed in retinal cell cultures maintained under hyperglycemia, as shown by increased mitochondrial fragmentation and reduced oxygen consumption rates (8). The accumulation of damaged mitochondria disrupts normal tissue homeostasis and leads to exacerbated oxidative stress, energy deficits, and eventual cell apoptosis (9).

The maintenance of a healthy mitochondrial network within cells depends upon mitochondrial quality control (MQC) mechanisms, which regulate the balance between mitophagy (autophagic removal of damaged mitochondria) and biogenesis (de novo synthesis of mitochondria) (10). Mitophagy typically occurs in damaged mitochondria upon dissipation of the mitochondrial membrane potential (ψm), leading to stabilization of Pink1 at the outer mitochondrial membrane (OMM). This primes mitochondria for autophagy via activation of the E3-ubiquitin ligase Parkin (11, 12). Mitochondrial biogenesis is a dynamic process that is regulated in response to cellular metabolic demands and increases following the induction of mitophagy (13). Although several effectors are involved, PGC-1α and mitochondrial transcription factor A (TFAM) are critical in driving the replication of mtDNA and synthesis of proteins encoded in its genome (13).

The disruption of MQC has recently been implicated as a major cause of neurovascular pathology in a number of neurodegenerative disorders, including Parkinson’s and Alzheimer’s diseases (14). Here, we show for the first time to our knowledge that mitophagy is dysregulated and uncoupled from mitochondrial biogenesis during the progression of DR.

## Results

### Mitochondrial contents change during the progression of DR in human and murine retinas.

**Figure 1:**
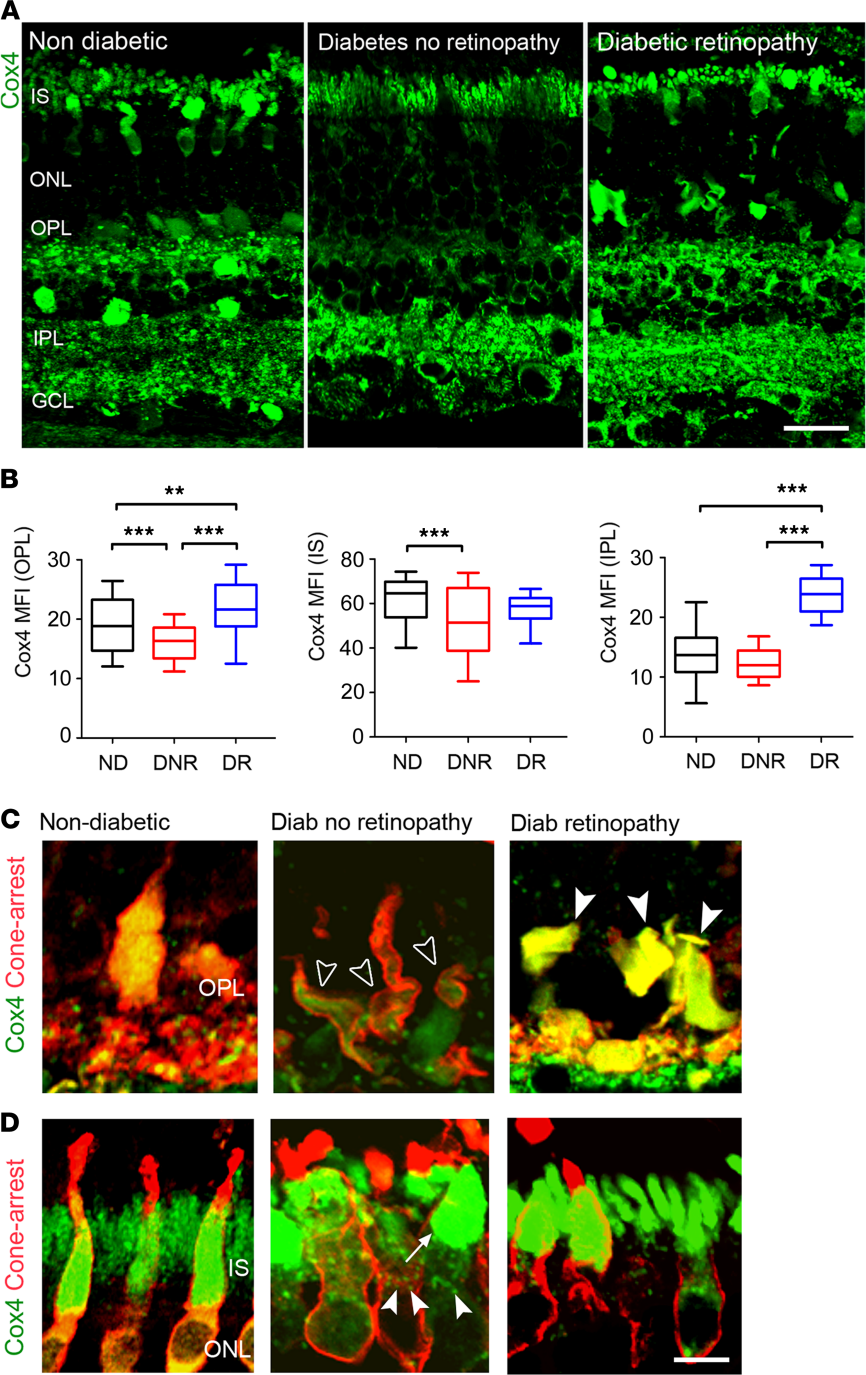
Cox4^+^ mitochondrial contents shift during the progression of diabetes in human retinas. (**A**) Retinal micrographs of human retinas from nondiabetic (ND), diabetic with no retinopathy (DNR), and diabetic retinopathy (DR) individuals processed for Cox4 immunostaining. (**B**) The mean fluorescence intensities (MFI) of Cox4 in photoreceptor IS, OPL, and IPL of ND (*n* = 3 eyes), DNR (*n* = 5 eyes), and DR (*n* = 2 eyes) individuals (*n* = 16 technical replicates per donor eye were used). Data are presented in box-and-whisker plots. (**C** and **D**) Retinal micrographs from ND, DNR, and DR individuals processed for Cox4 and cone arrestin immunostaining in (**C**) photoreceptor synaptic terminals and (**D**) photoreceptor IS. (**C**) Loss (open arrowheads) and gain (closed arrowheads) of mitochondrial contents in cone photoreceptor synaptic terminals. (**D**) Redistribution (arrow) and fragmentation (arrowheads) of Cox4^+^ mitochondria in cone photoreceptor IS. **P < 0.01, ***P < 0.001. One-way ANOVA with Bonferroni’s correction for multiple comparisons. IS, photoreceptor inner segments; ONL, outer nuclear layer; OPL, outer plexiform layer; IPL, inner plexiform layer; GCL, ganglion cell layer. Scale bars: 40 μm (**A**), 10 μm (**C** and **D**).

We first investigated mitochondrial contents in human retinas from nondiabetic (ND) subjects, from people with diabetes but no retinopathy (DNR), and from people with DR, using Cox4 antibody. Overall, compared with ND, Cox4 levels were lower in DNR but elevated in DR (Figure 1A). Quantitative analysis of Cox4 in specific retinal layers of DNR subjects revealed a significant reduction within synaptic processes of the outer plexiform layer (OPL) as compared with ND (Figure 1, B and C). Cox4 was also significantly reduced in the inner segments (IS) of cone photoreceptors in DNR (Figure 1B), further revealing mitochondrial morphological alterations in DNR subjects, including mitochondrial redistribution throughout the IS (Figure 1D) and abnormal fragmentation (Figure 1D). No changes were observed in Cox4 levels at the inner plexiform layer (IPL) of DNR (Figure 1B). Interestingly, Cox4 contents were not reduced in DR subjects, displaying instead a significant increase in OPL and IPL (Figure 1, A–C).

**Figure 2:**
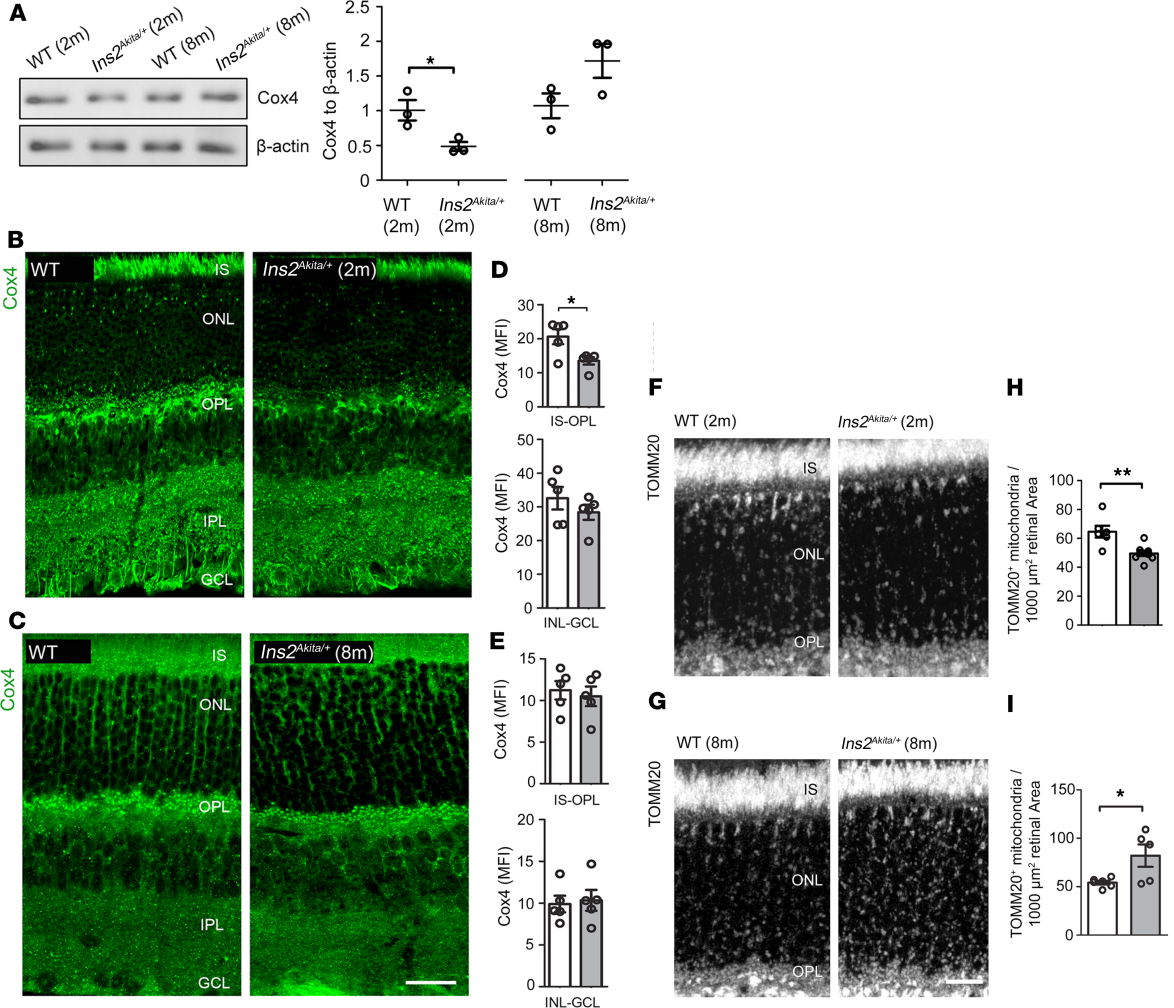
Mitochondrial contents shift during the progression of diabetes in *Ins2^Akita/+^* mouse retinas. (**A**) Example immunoblot and quantification of Cox4 in retinal lysates of 2-month and 8-month hyperglycemic *Ins2^Akita/+^* and age-matched WT mice. Data were normalized to β-actin loading controls. (**B** and **C**) Retinal micrographs of 2-month (**B**) and 8-month (**C**) hyperglycemic *Ins2^Akita/+^* and age-matched WT mice processed for Cox4 immunostaining. (**D** and **E**) The mean fluorescence intensities (MFI) of Cox4 at the IS-OPL and INL-GCL of 2-month (**D**) and 8-month (**E**) hyperglycemic *Ins2^Akita/+^* and age-matched WT mice. (**F** and **G**) Retinal micrographs of 2-month (**F**) and 8-month (**G**) hyperglycemic *Ins2^Akita/+^* and age-matched WT mice processed for TOMM20 immunostaining. (**H** and **I**) The densities of TOMM20^+^ mitochondria at the IS-OPL of 2-month (**H**) and 8-month (**I**) hyperglycemic *Ins2^Akita/+^* and age-matched WT mice. WT (white bars), *Ins2^Akita/+^* (gray bars); *n* = 5–8 eyes per strain. Results presented as mean ± SEM. *P < 0.05, **P < 0.01, 2-sided unpaired Student’s *t* test. IS, photoreceptor inner segments; ONL, outer nuclear layer; OPL, outer plexiform layer; IPL, inner plexiform layer; GCL, ganglion cell layer. Scale bar: 40 μm (**B** and **C**), 20 μm (**G**).

To provide a basis for better understanding why mitochondrial contents shift during the course of diabetes, we examined whether these changes are recapitulated in a preclinical model of type-1 diabetes. Cox4 levels were investigated in 2- and 8-month-old hyperglycemic *Ins2^Akita/+^* mice, which exhibit mild to severe retinal neurovascular dysfunction at these time points, respectively (15–17). Cox4 immunoblots revealed loss of mitochondrial contents in 2-month hyperglycemic *Ins2^Akita/+^* mice (Figure 2A). Immunohistochemical analysis revealed a specific decrease of Cox4 at the outer (IS-OPL) but not inner retinal layers (from inner nuclear [INL] to ganglion cell layer [GCL]; Figure 2, B and D). In contrast, Cox4 contents were unaffected in 8-month-old hyperglycemic *Ins2^Akita/+^* mice (Figure 2, A, C, and E). This change of mitochondrial contents at the outer retina of *Ins2^Akita/+^* mice included photoreceptors (as assessed specifically in IS and OPL layers; Supplemental Figure 1; supplemental material available online with this article; https://doi.org/10.1172/jci.insight.129760DS1) and Müller cells, given the enrichment of mitochondria within glutamine synthase–positive processes across the ONL (Supplemental Figure 2). Taken together, our data suggest that mitochondrial contents decline at the outer retina of human and *Ins2^Akita/+^* mice at the early stages of diabetes but increase during the development of DR. This was clearly established using immunostaining against TOMM20 (a translocator of the OMM), which delineated the whole mitochondrial network at the outer retina (Figure 2, F–I).Moreover, no changes of Cox4 mRNA levels (*Cox4i1* and *Cox4i2* isoforms) were detected in 2-month and 8-month-old hyperglycemic *Ins2^Akita/+^* retinas (as compared with age-matched controls, Supplemental Figure 3), suggesting that mitochondrial changes occur due to an altered mitochondrial turnover in diabetes.

### Exacerbated mitophagy occurs in Ins2^Akita/+^ retinas at early stages of diabetes.

To determine why mitochondrial contents are reduced at the early stages of diabetes, we investigated mitochondrial biogenesis and mitophagy in 2-month-old hyperglycemic *Ins2^Akita/+^* retinas. Mitochondrial biogenesis was evaluated by assessing 2 of the main effectors that regulate mtDNA transcription — namely, PGC-1α and TFAM (13). No significant changes in the protein levels of PGC-1α were observed in 2-month-old hyperglycemic *Ins2^Akita/+^* retinas (Supplemental Figure 4, A–C). TFAM immunostaining in WT mice revealed enrichment of mitochondrial nucleoids (where mtDNA is packaged into discrete mtDNA-protein complexes) (18) throughout the retina, but no significant changes in their density were observed at the IS-OPL (Supplemental Figure 4, D–F). In addition, no mtDNA damage (Supplemental Figure 4G) or variations in mtDNA copy number (Supplemental Figure 4H) were detected, supporting the absence of changes in mitochondrial biogenesis.

**Figure 3:**
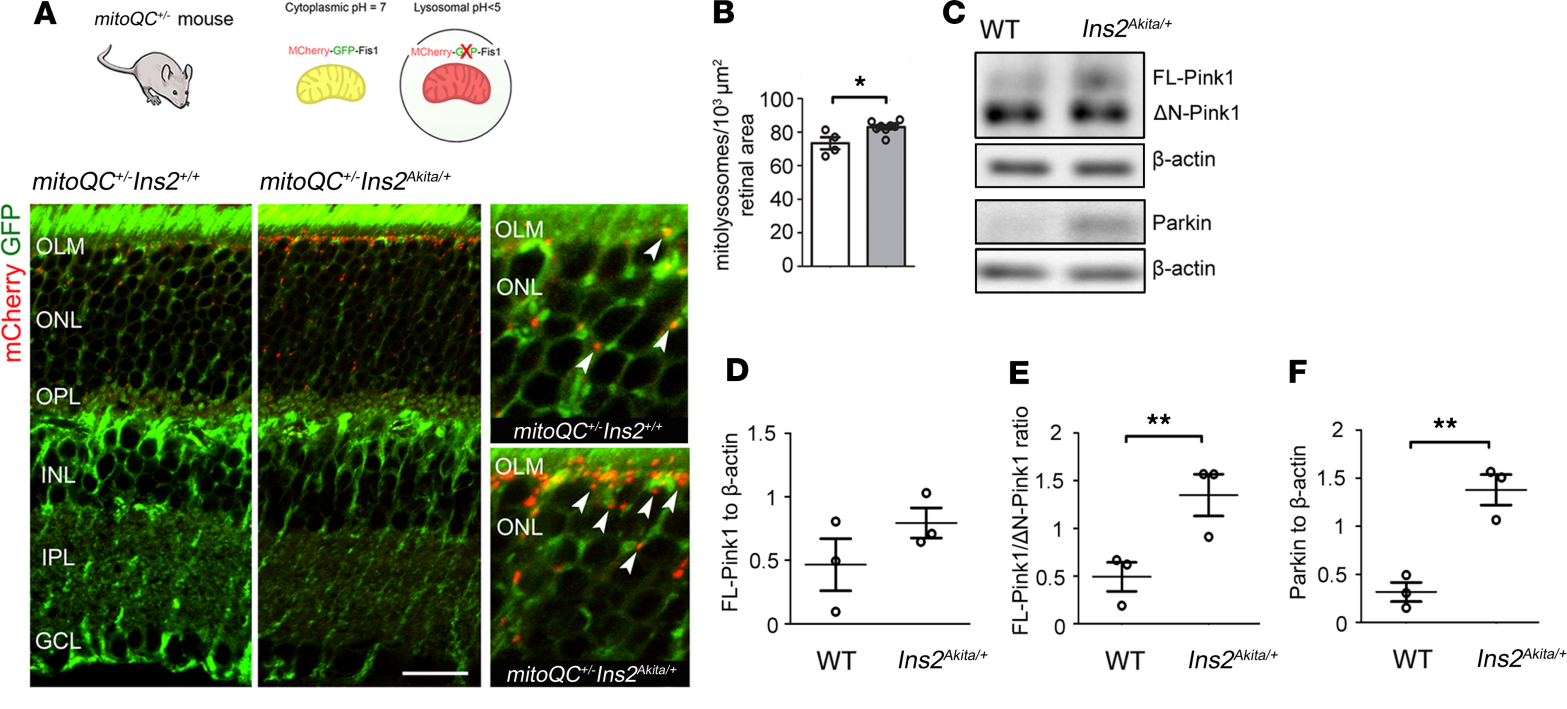
Increased mitophagy at the outer retina of 2-month hyperglycemic *Ins2^Akita/+^* mice. (**A**) Confocal photomicrographs showing mitolysosomes (mCherry-only foci, arrowheads) at the IS-OPL of 2-month hyperglycemic mitophagy reporter mice (*mitoQC^+/–^ Ins2^Akita/+^*) and nondiabetic siblings (*mitoQC^+/–^ Ins2^+/+^*). (**B**) Mitolysosome density at the IS-OPL. *mitoQC^+/–^ Ins2^+/+^* (white bars), *mitoQC^+/–^ Ins2^Akita/+^* (gray bars). (**C–F**) Example immunoblot (**C**) and quantification of Pink1-dependent mitophagy proteins in retinal lysates (**D–F**) of 2-month hyperglycemic *Ins2^Akita/+^* and age-matched WT mice. Data were normalized to β-actin loading controls. *n* = 3–7 eyes per strain. Results presented as mean ± SEM. *P < 0.05, **P < 0.01, 2-sided unpaired Student’s *t* test. IS, photoreceptor inner segments; OLM, outer limiting membrane, ONL, outer nuclear layer; OPL, outer plexiform layer; INL, inner nuclear layer; IPL, inner plexiform layer; GCL, ganglion cell layer. Scale bar: 40μm

To unambiguously investigate mitophagy in the diabetic retina, we generated *Ins2^Akita^* mitophagy-reporter mice (*mitoQC^+/–^ Ins2^Akita/+^*), by mating *mitoQC^+/+^* (19) with *Ins2^Akita/+^* mice (Figure 3A). In line with a recent report (19), mitophagy was mostly detected at the outer retina, as verified by high mitolysosome density (mCherry-only foci) in the IS-OPL of ND *mitoQC^+/–^ Ins2^+/+^* mice (Figure 3A). Diabetes amplified mitophagy at the outer retina, as indicated by a significant increase of mitolysosomes in 2-month hyperglycemic *mitoQC^+/–^ Ins2^Akita/+^* mice (Figure 3, A and B). These findings were further supported by analysis of Pink1 (a primary effector for the autophagic degradation of mitochondria in lysosomes; ref. 11) in retinal lysates (Figure 3, C–F). In healthy polarized mitochondria, Pink1 (full-length Pink1; FL-Pink1) shuttles to the mitochondrial matrix and is rapidly cleaved by presenilin-associated rhomboid-like protein (PARL) into N-terminal–cleaved Pink1 (ΔN-Pink1) (20). However, upon dissipation of Ψm, the internalization of FL-Pink1 within mitochondria is prevented, and it stabilizes at the OMM, triggering the onset of mitophagy. Consistent with the increased levels of mitophagy in diabetic retinas, Pink1 levels were shifted toward its immature form, as shown by a significant elevation in the FL-Pink1/ΔN-Pink1 ratio (Figure 3, C–E). Further validation was carried out by immunohistochemical approaches. As an index of Pink1-dependent mitophagy, we assessed the percentage of Pink1^+^ puncta colocalizing with LAMP1^+^ lysosomes, and it was found to increase in the outer retina of 2-month hyperglycemic *Ins2^Akita/+^* mice (Supplemental Figure 5). Mitophagy depends on an efficient autophagic flux, and this appeared to be increased at the outer retina of 2-month-old hyperglycemic *Ins2^Akita/+^* mice (based on similar Lc3b^+^ autophagosomes but reduced levels of the autophagy substrate p62/SQTSM1; Supplemental Figure 6, A, B, E, F, I, and J) (21). Overall, our data strongly suggest that exacerbated Pink1-dependent mitophagy (together with normal mitochondrial biogenesis) underlies the reduction of mitochondrial contents at the outer retina of *Ins2^Akita/+^* mice during the early stages of diabetes.

### Mitochondrial biogenesis fails to compensate for hyperglycemia-induced mitophagy in cultured retinal Müller cells.

**Figure 4:**
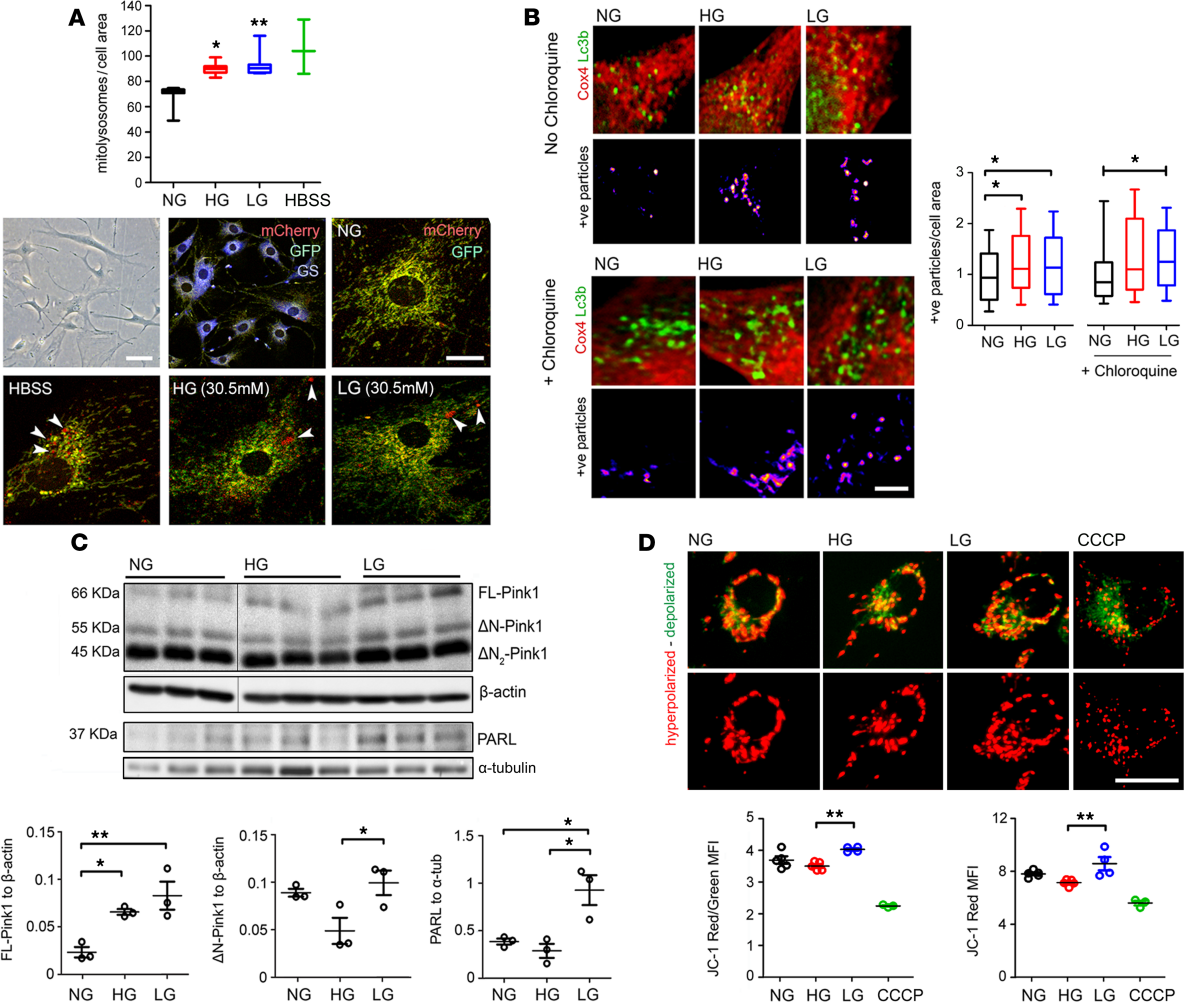
The diabetic milieu dysregulates mitophagy in primary Müller and MIO-M1 cultures in vitro. (**A–D**) Primary retinal Müller cells isolated from *mitoQC^+/+^* mouse (*mitoQC*-PMCs) (**A**) or human MIO-M1 cells (**B–D**) were maintained for 5 days in normal glucose (NG, 5.5mM), high glucose (HG, 30.5mM), or L-glucose (LG, 30.5 mM) osmotic control. (**A**) *mitoQC*-PMCs had a flattened-elongated shape (bright-field image) and were positive for glutamine synthase (GS) immunoreactivity. Mitolysosome density (mCherry-only foci, arrowheads) was evaluated as index of mitophagy flux. Positive controls were established following amino acid starvation with HBSS (16 hours). Data are presented in box-and-whisker plots; *n* = 3–5 biological replicates per group. (**B**) Quantification of Cox4/Lc3b colocalizing particles in different treatment groups ± 100 μM chloroquine for final 12 hours of treatment. Data presented as fold-change vs. NG control cells in box-and-whisker plots; at least 70 cells are included, obtained from *n* = 3 biological replicates per group. (**C**) Example immunoblot and quantification of Pink1-dependent mitophagy proteins in different treatment groups. Data were normalized to β-actin or α-tubulin loading controls; *n* = 3 biological replicates per group. Pink1 lanes and corresponding β-actin loading controls were run on the same gel but were noncontiguous. (**D**) Evaluation of mitochondrial membrane potential by JC-1 dye (red, hyperpolarized mitochondria; green, depolarized mitochondria) in different treatment groups. CCCP (20 μM) was added as a mitochondrial uncoupler positive control (16 hours); *n* = 3–4 biological replicates per group. Results presented as mean ± SEM in **A**, **C**, and **D**. *P < 0.05, **P < 0.01. One-way ANOVA with Bonferroni’s correction for multiple comparisons; MFI, mean fluorescence intensity. Scale bar: 100 μm (**A**, bright-field), 20 μm (**A**, mCherry-GFP; **D**), 2 μm (**B**).

To determine in detail how mitochondrial turnover is affected by the diabetic milieu, the interplay between mitophagy and mitochondrial biogenesis was investigated under hyperglycemic conditions in vitro. Müller cells were selected for this study given (a) the predominant role of mitochondrial oxidative phosphorylation to maintain Müller basal functions (22), (b) the recognized importance of Müller cells in the pathogenesis of DR (23), and (c) the uniform distribution of mitochondria in Müller cells throughout the entire thickness of the neuroretina (24). To address mitophagy, we took advantage of primary Müller cells (PMCs) isolated from *mitoQC* mice. The purity of *mitoQC*-PMCs was confirmed by glutamine synthase immunostaining (Figure 4A) (25). The induction of mitophagy in *mitoQC*-PMCs was initially validated by HBSS amino acid starvation, which significantly exacerbated the density of mitolysosomes (Figure 4A) (26). *MitoQC-*PMCs maintained in high glucose (HG; 30.5 mM D-glucose) showed a significant increase of mitolysosomes, as compared with normal glucose (NG; 5.5 mM D-glucose) controls (Figure 4A). Interestingly, mitophagy was also elicited by hyperosmolar changes, as observed in 30.5 mM L-glucose (LG) cultures (Figure 4A). The induction of mitophagy by HG and LG was corroborated in the human Müller cell line Moorfields/Institute of Ophthalmology- Müller 1 (MIO-M1) by colocalization analysis of Cox4 with Lc3b^+^ autophagosomes (26) (Figure 4B). MIO-M1 cultures maintained under HG or LG for 5 consecutive days exhibited a significant increase in Cox4/Lc3b colocalization (Figure 4B), suggestive of increased mitophagy. To further substantiate this result, chloroquine was used to block autophagosome fusion with lysosomes (thus allowing the accumulation of mitochondria within autophagosomes). In line with an increased mitophagy flux, chloroquine further exacerbated the density of Cox4/Lc3b colocalizing particles in HG and LG MIO-M1 cultures (as compared with NG chloroquine-treated cultures; Figure 4B).

Since our in vivo data suggest a role for Pink1 in diabetes-induced mitophagy (Figure 3, C–E), we next evaluated the involvement of this pathway in HG- and LG-mediated mitophagy. Suggestive of increased Pink1-dependent mitophagy, MIO-M1 cultures maintained in HG showed a significant stabilization of FL-Pink1 (Figure 4C) and a significant elevation in the FL-Pink1/ΔN-Pink1 ratio (data not shown). Similar results were found in MIO-M1 cultures maintained in LG (Figure 4C). Interestingly, the levels of the cleaved product ΔN-Pink1 were significantly higher in LG when compared with HG cultures (Figure 4C). This result led us to hypothesize that ΔN-Pink1 levels in LG may remain constant via an increase in the number of hyperpolarized mitochondria and upregulation of PARL (which would allow the steady cleavage of FL-Pink1 within the mitochondrial matrix). As suggested, the contents of PARL (Figure 4C) and hyperpolarized mitochondria (JC-1 red fluorescence, Figure 4D) were increased in LG MIO-M1 cultures. By contrast, HG cultures, which showed a decrease in ΔN-Pink1, lacked such compensation, as reflected by the unchanged contents of PARL and hyperpolarized mitochondria (Figure 4, C and D).

**Figure 5:**
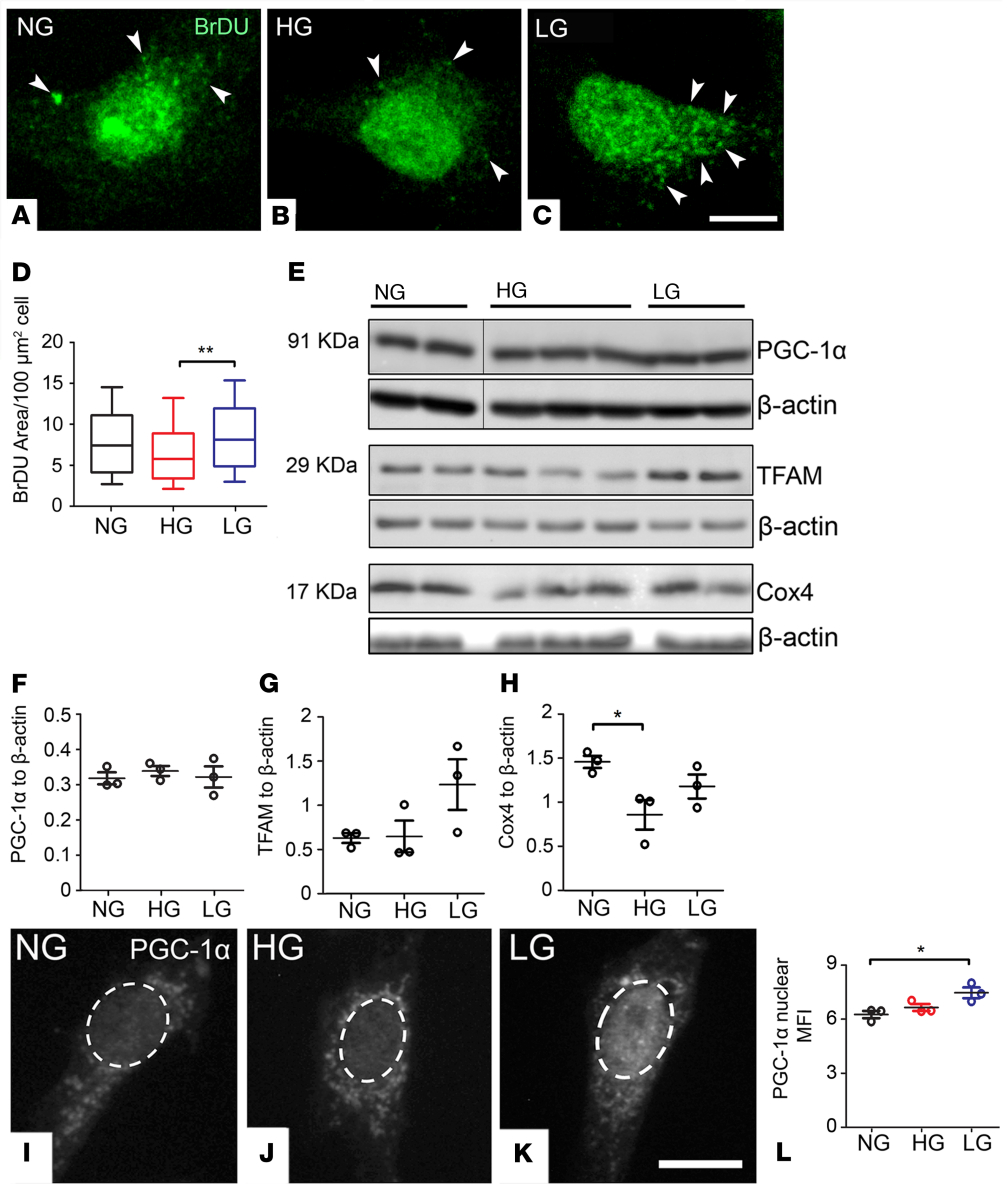
The diabetic milieu dysregulates mitochondrial biogenesis in MIO-M1 cultures in vitro. MIO-M1 cells were maintained for 5 days in normal glucose (NG, 5.5 mM), high glucose (HG, 30.5 mM), or L-glucose (LG, 30.5 mM) osmotic control. (**A–D**) Representative confocal micrographs (**A–C**) and quantification (**D**) of mitochondrial biogenesis by incorporation of Bromodeoxyuridine (BrDU) into mtDNA (arrowheads) in different treatment groups; data are presented in box-and-whisker plots. At least 70 cells were used, obtained from *n* = 3 biological replicates per group. (**E–H**) Example immunoblot (**E**) and quantification (**F–H**) of mitochondrial biogenesis proteins in different treatment groups. Data were normalized to β-actin loading control; *n* = 3 biological replicates per group. PGC-1α lanes and corresponding β-actin loading controls were run on the same gel but were noncontiguous. PGC-1α shared similar β-actin loading controls to those in Figure 4C (Pink1). (**I–K**) Representative confocal micrographs of PGC-1α immunostaining in different treatment groups. (**L**) Quantification of nuclear PGC-1α mean fluorescence intensity (MFI) in different treatment groups; *n* = 3 biological replicates per group. Results presented as mean ± SEM in **F–H** and **L**. *P < 0.05, **P < 0.01. One-way ANOVA with Bonferroni’s correction for multiple comparisons. Scale bars: 10 μm.

The above results suggest that HG and LG accelerate mitophagy but only in the case of LG is this compensated by an increase in mitochondrial biogenesis. This idea was tested further by studying mitochondrial biogenesis. BrDU incorporation into mtDNA (comprising the gold-standard for evaluating mitochondrial biogenesis) (27) was unchanged in MIO-M1 cultures subjected to HG (Figure 5, A, B, and D). This was validated at the molecular level, since the contents of molecular adaptors controlling mitochondrial biogenesis, including PGC-1α (total, Figure 5, E and F; nuclear, Figure 5, I, J, and L) and TFAM (Figure 5, E and G), remained unaltered. In comparison with HG, LG cultures exhibited increased mitochondrial biogenesis, as shown by the increased incorporation of BrDU into mtDNA (Figure 5, A–D), upregulated levels of nuclear PGC-1α (Figure 5, I–L), and an apparent increase of TFAM (Figure 5, E and G).

To confirm differences in the balance between mitochondrial biogenesis and mitophagy, we assessed the relative age of mitochondria. MIO-M1 cultures were transfected with *pMitoTimer* (28), and the ratio of red (old) vs. green (young) fluorescent mitochondria was determined (the smaller the ratio, the younger mitochondrial network). Compared with HG, LG cultures were expected to have younger mitochondrial populations, due to accelerated mitochondrial synthesis/degradation. Accordingly, LG but not HG cultures exhibited a significant decrease in *pMitoTimer* red/green (R/G) ratio (Supplemental Figure 7). Collectively, these data suggest that HG activates mitophagy through a pathway involving hyperosmotic stress. In contrast, the inability of mitochondrial biogenesis to compensate for increased HG-induced mitophagy appears to result from a metabolic rather than hyperosmotic effect. These findings may explain the loss of mitochondrial contents observed in the diabetic retina (Figure 1 and Figure 2) and in MIO-M1 cultures maintained under hyperglycemia (Cox4 immunoblots; Figure 5, E and H).

### Mitophagy is impaired in Ins2^Akita/+^ retina at advanced stages of neurovascular dysfunction.

**Figure 6:**
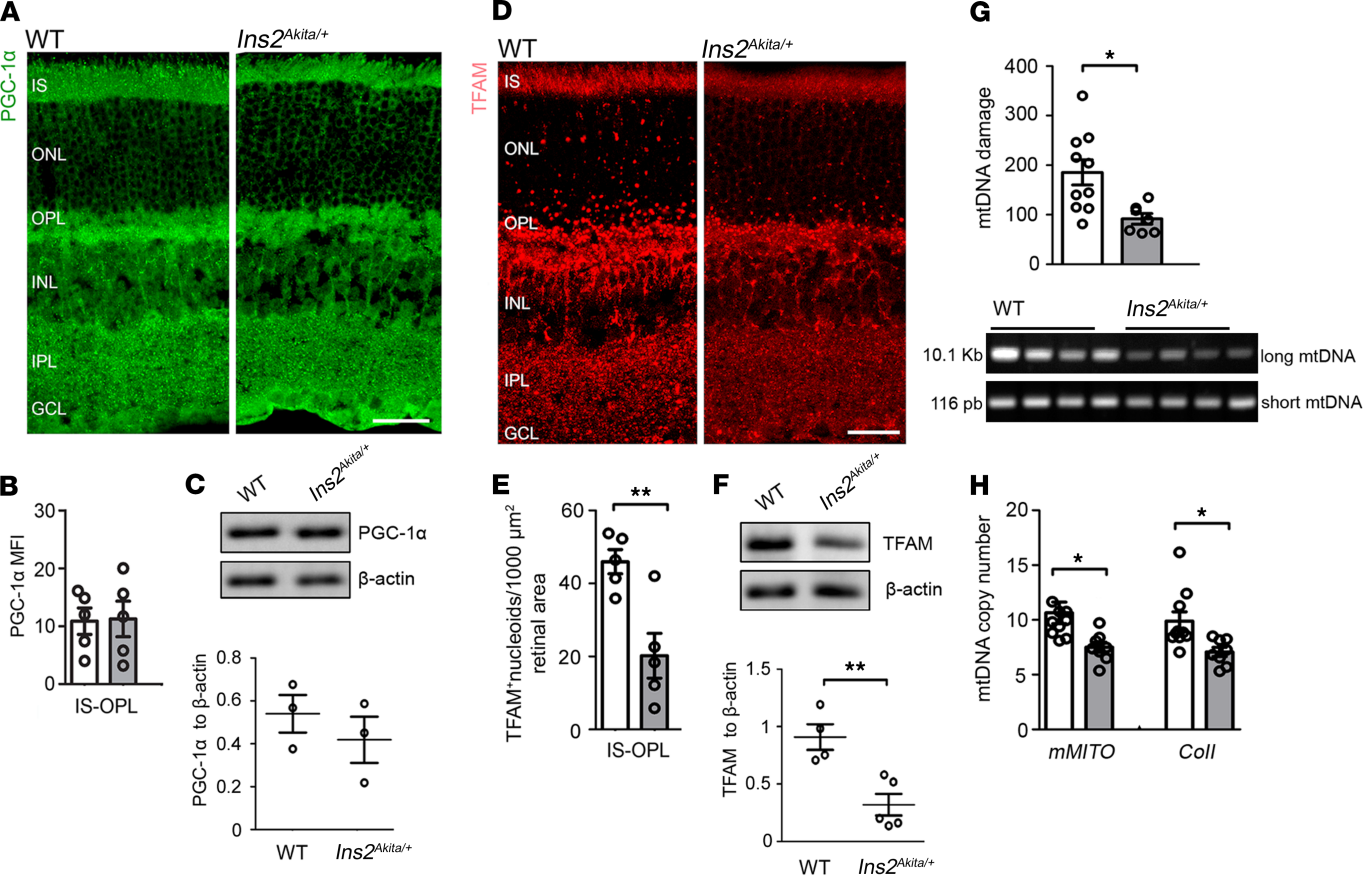
Impairment of mitochondrial biogenesis machinery in 8-month hyperglycemic *Ins2^Akita/+^* mouse retinas. (**A**) Retinal micrographs from 8-month hyperglycemic *Ins2^Akita/+^* and age-matched WT mice processed for PGC-1α immunostaining. (**B**) The mean fluorescence intensity (MFI) of PGC-1α at the IS-OPL. (**C**) Example immunoblot and quantification of PGC-1α in mouse retinal lysates of 8-month hyperglycemic *Ins2^Akita/+^* and age-matched WT. (**D**) Retinal micrographs from 8-month hyperglycemic *Ins2^Akita/+^* and age-matched WT mice processed for TFAM immunostaining. (**E**) The density of TFAM^+^ mitochondrial nucleoids at the IS-OPL. (**F**) Example immunoblot and quantification of TFAM in mouse retinal lysates of 8-month hyperglycemic *Ins2^Akita/+^* and age-matched WT. Data were normalized to β-actin loading controls. TFAM shared similar β-actin loading controls to those in Figure 2A (Cox4). (**G**) Evaluation of mtDNA damage in 8-month hyperglycemic *Ins2^Akita/+^* and age-matched WT mouse retinas by amplification of long (10.1 Kb) and short (116 pb) mtDNA regions. A reduction in the long/short amplification ratio is indicative of mtDNA damage. (**H**) Mitochondrial copy numbers evaluated by real-time PCR analysis of *mMITO* and *CoII* mtDNA regions. WT (white bars), *Ins2^Akita/+^* (gray bars); *n* = 3–10 eyes per strain. Results presented as mean ± SEM. *P < 0.05, **P < 0.01, 2-sided unpaired Student’s *t* test. IS, photoreceptor inner segments; ONL, outer nuclear layer; OPL, outer plexiform layer; INL, inner nuclear layer; IPL, inner plexiform layer; GCL, ganglion cell layer. Scale bars: 40μm.

To understand the shift toward increasing mitochondrial contents at advanced stages of DR (Figure 1 and Figure 2), we investigated mitochondrial biogenesis and mitophagy in 8-month-old hyperglycemic *Ins2^Akita/+^* mice. In contrast to younger ages, the mitochondrial biogenesis machinery was shown to be impaired at this stage, as indicated by a decrease in TFAM protein levels and of TFAM^+^ mitochondrial nucleoids at the IS-OPL (Figure 6, A–F), substantial mtDNA damage (Figure 6G), and reduced mtDNA copy number (Figure 6H). Upregulated mitochondrial biogenesis cannot, therefore, explain the normalization of Cox4 levels observed in 8-month-old hyperglycemic *Ins2^Akita/+^* mice.

**Figure 7:**
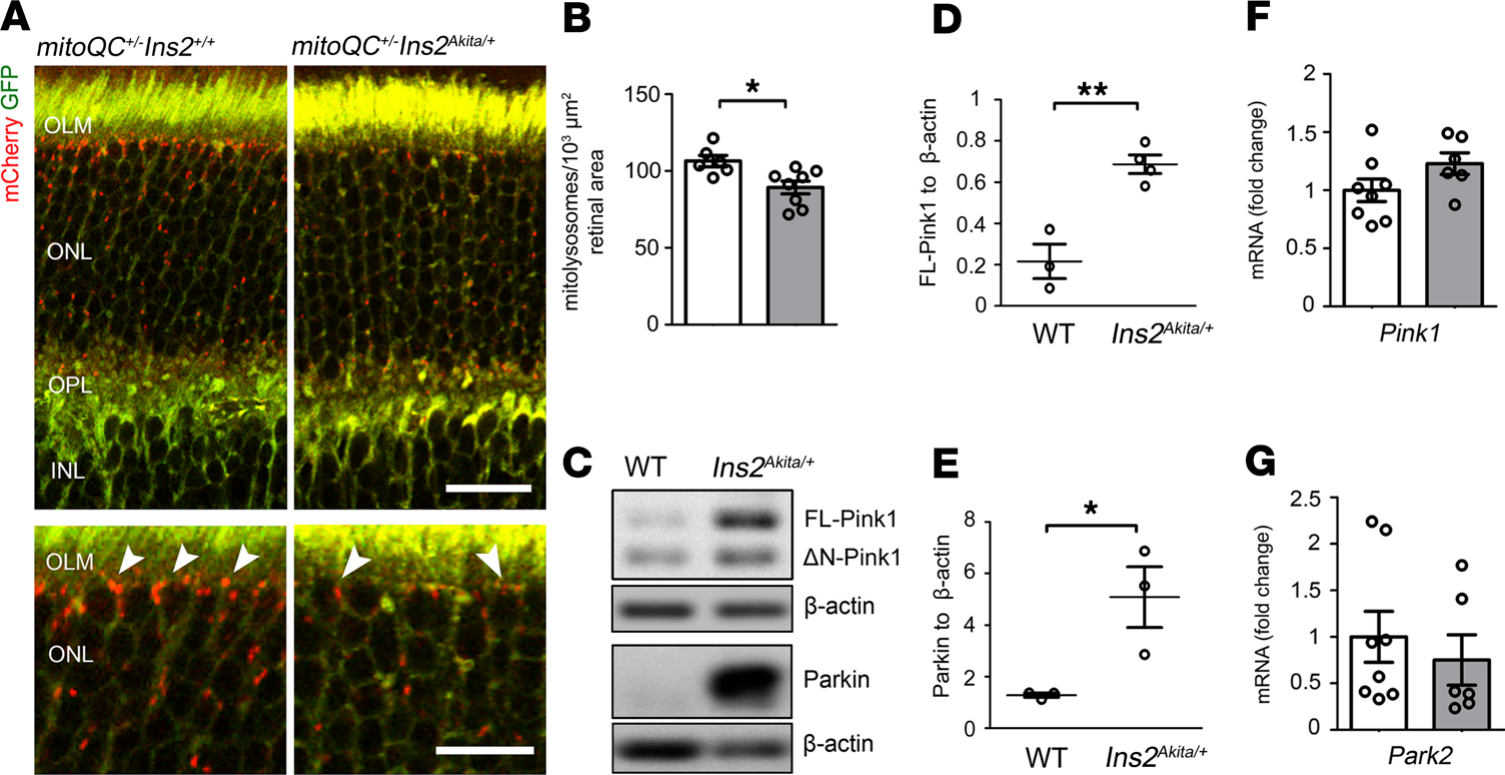
Mitophagy is decreased at the outer retina of 8-month hyperglycemic *Ins2^Akita/+^* mice. (**A**) Confocal photomicrographs showing mitolysosomes (mCherry-only foci, arrowheads) at the IS-OPL of 8-month hyperglycemic mitophagy reporter mice (*mitoQC^+/–^ Ins2^Akita/+^*) and nondiabetic siblings (*mitoQC^+/–^ Ins2^+/+^*). (**B**) Mitolysosome density at the IS-OPL. *mitoQC^+/–^ Ins2^+/+^* (white bars), *mitoQC^+/^ Ins2^Akita/+^* (grey bars). (**C–E**) Example immunoblot (**C**) and quantification (**D** and **E**) of Pink1-dependent mitophagy proteins in retinal lysates of 8-month hyperglycemic *Ins2^Akita/+^* and age-matched WT mice. Data were normalized to β-actin loading controls. (**F** and **G**) Real-time PCR analysis of *Pink1* and *Park2* gene transcripts in the retina of 8-month hyperglycemic *Ins2^Akita/+^* and age-matched WT mice. WT (white bars), *Ins2^Akita/+^* (gray bars); *n* = 3–8 eyes per strain. Results presented as mean ± SEM. *P < 0.05, **P < 0.01, 2-sided unpaired Student’s *t* test. IS, photoreceptor inner segments; OLM, outer limiting membrane; ONL, outer nuclear layer; OPL, outer plexiform layer; INL, inner nuclear layer. Scale bars: 40 μm, 20 μm (inset).

Importantly, the analysis of mitophagy revealed a significant decrease of mitolysosomes at the outer retina of 8-month-old hyperglycemic *mitoQC^+/–^ Ins2^Akita/+^* mice (as compared with ND *mitoQC^+/–^ Ins2^+/+^*, Figure 7, A and B). However, FL-Pink1 stabilization and Parkin levels were strikingly increased (Figure 7, C–E), suggesting that Pink1-primed mitochondria are ineffectively cleared and accumulate in the retina at advanced stages of diabetes. This was further evidenced by a significant accumulation of ubiquitin and p62 in the mitochondria of photoreceptor IS; however, this was not observed in younger diabetic stages with competent mitophagy (Supplemental Figure 8). Autophagy adaptors were also found to accumulate at the outer retina, indicated by increased Lc3b^+^ autophagosomes and p62/SQTSM1 (Supplemental Figure 6, C, D, and G–J). Importantly, the gene transcripts of those mitophagy (*Pink1*, *Park2*) (Figure 7, F and G) and autophagy (*Map1lc3b*, *sqstm1*) (Supplemental Figure 6K) effectors were unchanged in the diabetic retina at this stage, suggesting that their accumulation at the protein level was due to inefficient autophagy/mitophagy. To confirm this hypothesis, we evaluated the levels of mitochondria entering (a) autophagosomes (Cox4/Lc3b colocalization) and (b) Pink1-dependent mitophagy (Pink1/LAMP1 colocalization). The levels of mitochondria colocalizing with Lc3b^+^ autophagosomes were significantly increased at the outer retina (Supplemental Figure 9, A and C); however, their degradation by lysosomes appeared impaired as the levels of Pink1 colocalizing with LAMP1 were not elevated (Supplemental Figure 9, B and D). Thus, disruption of Pink1 mitophagy may contribute to the build-up of mitochondria primed for degradation during advanced stages of DR.

### Disruption of mitophagy in the diabetic retina is associated with increased cellular senescence.

**Figure 8:**
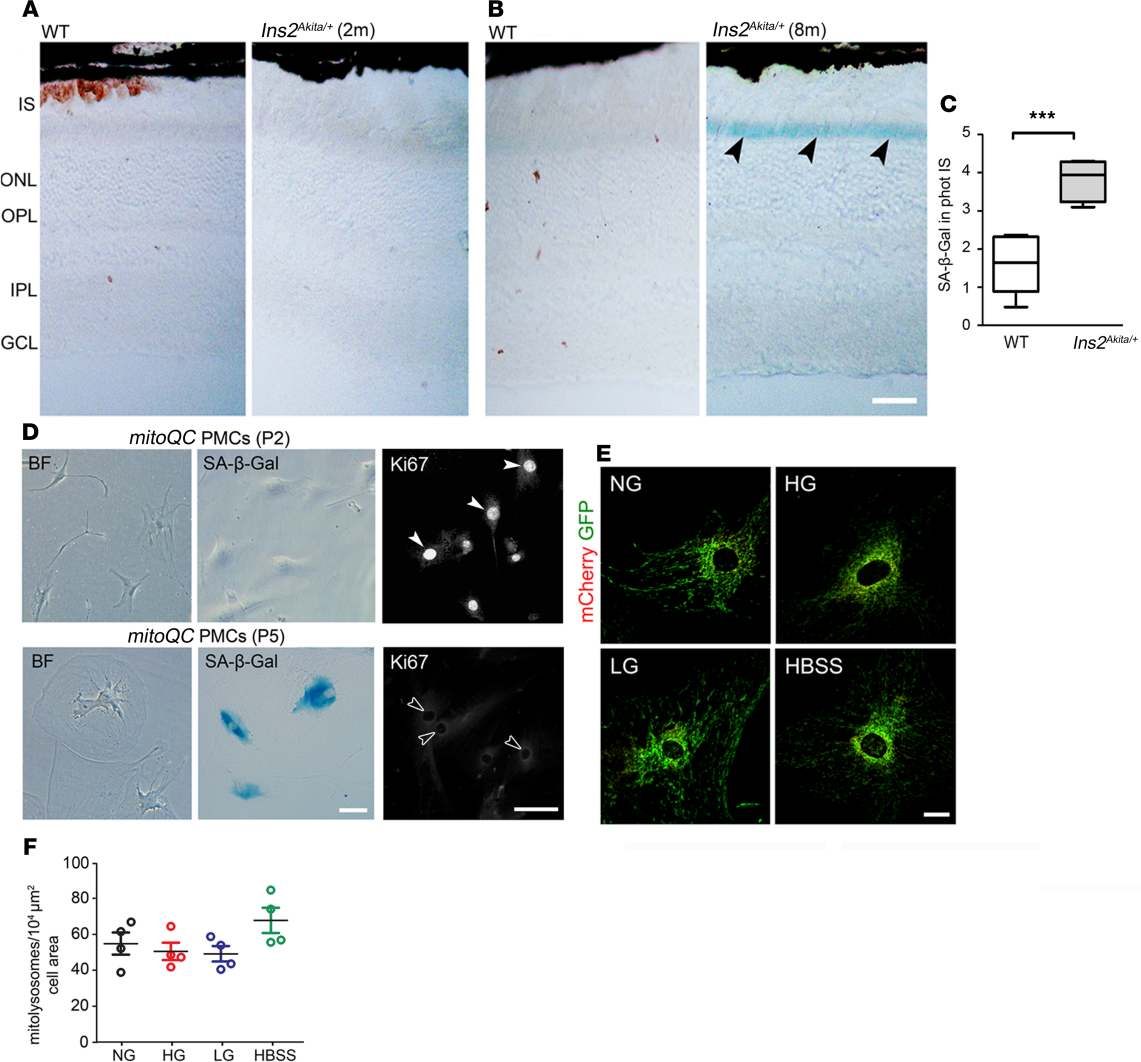
Senescence in the diabetic retina may disrupt mitochondrial quality control. (**A** and **B**) Retinal micrographs from 2-month (**A**) and 8-month (**B**) hyperglycemic *Ins2^Akita/+^* and age-matched WT mice processed for SA-β-Gal activity. Increased SA-β-Gal activity in photoreceptor IS of 8-month hyperglycemic *Ins2^Akita/+^* mice (arrowheads). (**C**) The levels of SA-β-Gal activity in photoreceptor IS in 8-month hyperglycemic *Ins2^Akita/+^* and age-matched WT. Data are presented in box-and-whisker plots; *n* = 6 eyes per group. (**D**) Bright-field (BF) images of primary retinal Müller cells isolated from *mitoQC^+/+^* mouse (*mitoQC*-PMCs) showing their morphology, SA-β-Gal activity, and Ki67 immunostaining at passage 2 (P2) and P5. Proliferative nonsenescent *mitoQC*-PMCs show high nuclear-levels of Ki67 (P2, closed arrowheads), in contrast to senescent cultures (P5, open arrowheads). (**E** and **F**) Mitolysosome (mCherry-only foci) density was quantified in P5–P6 *mitoQC* PMCs maintained for 5 days in normal glucose (NG, 5.5 mM), high glucose (HG, 30.5 mM), or L-glucose (LG, 30.5 mM) osmotic control. Amino acid starvation with HBSS (16 hours) does not elicit mitophagy in senescent mitoQC PMCs; *n* = 2 biological replicates and 4 technical replicates per group. Results are presented as mean ± SEM. ***P < 0.001, 2-sided unpaired Student’s *t* test in **C**; 1-way ANOVA with Bonferroni’s correction for multiple comparisons in **F**. IS, photoreceptor inner segments; ONL, outer nuclear layer; OPL, outer plexiform layer; INL, inner nuclear layer; IPL, inner plexiform layer; GCL, ganglion cell layer. Scale bars: 40 μm (**A** and **B**), 20 μm (**D** and **E**).

Mitophagy is known to be perturbed in senescent cells, which may result in the accumulation of damaged mitochondria (29, 30). Hence, we finally investigated whether the impairment of mitophagy at advanced stages of diabetes is associated with a senescent retinal phenotype. In support of this, upregulated senescence-associated β-galactosidase (SA-β-Gal) activity (31) was observed at the outer retina of 8-month-old but not 2-month-old hyperglycemic *Ins2^Akita/+^* mice (Figure 8, A–C). To further understand whether diabetes-induced mitophagy may be impaired by cellular senescence, replicative, nonchemical senescence was induced in *mitoQC*-PMCs (by continuous passage [P] of the cells until P5–P6) and mitophagy elicited by HG, LG, or HBSS. Senescence was confirmed in P5–P6 *mitoQC*-PMCs cultures, as shown by exacerbated SA-β-Gal activity, an enlarged/flat morphology, and negligible nuclear levels of the proliferative marker Ki67 (Figure 8D). In contrast to earlier passages, mitophagy was not elicited in senescent *mitoQC*-PMCs following treatments with HG, LG, or HBSS (Figure 8, E and F). Hence, the premature senescence of cells at the outer retina may explain the disruption of mitophagy at advanced stages of diabetes.

## Discussion

Our study has demonstrated how MQC becomes dysregulated in the retina during the progression of diabetes. Early stages were characterized by the net loss of mitochondrial contents at the outer retina, as mitochondrial biogenesis was unable to compensate for increased diabetes-induced mitophagy. However, mitophagy was shown to decline with diabetes duration, shifting mitochondrial contents toward normal values. Our data further suggest that mitophagy may be disrupted by senescence at advanced stages of diabetes.

A key observation from our study involves the increased mitophagy of the diabetic retina, which particularly affected outer retinal layers. As observed for MIO-M1 cultures maintained under LG, the increased mitophagy of the diabetic retina may arise due to increased hyperosmotic stress. Although the mechanisms remain unclear, changes in cellular osmolarity have been reported to influence the turnover of mitochondria (32). Nonetheless, additional insults might also be responsible for the dysregulation of mitophagy in the retina at the early stages of diabetes. Photoreceptors are highly metabolic cells, exhibiting the highest contents of mitochondria in the retina (as they rely on oxidative phosphorylation to support vision) (33). Since this process generates large amounts of ROS capable of damaging mitochondria, a greater demand for ROS-mediated mitophagy (34) at the outer retina might be expected. Previous studies have demonstrated increased mitochondrial superoxide production in the diabetic retina (35), which, in turn, could exacerbate the mitophagy demands. The overproduction of mitochondrial ROS in cell cultures exposed to hyperglycemia is also well documented (36, 37). Further studies are therefore warranted to more precisely understand the factors that contributed to increased mitophagy in the diabetic retina.

Our study suggests that Pink1 may contribute to retinal mitophagy; however, as recently suggested, the rate of mitophagy in the retina was shown to be unaffected in *Pink1^–/–^* mice (19). Although this highlights a dispensable role for Pink1 in physiological conditions, our study supports an important role of this pathway to drive mitophagy in the diabetic context. Nonetheless, other pathways, including BNIP3L/NIX or TXNIP, might also be important for orchestrating mitophagy in diabetes (36, 38). Further investigations are thus needed to precisely map the importance of the different mitophagy pathways in the diabetic retina, which extends beyond the scope of this current study.

As shown in *Ins2^Akita/+^* mice and in hyperglycemic MIO-M1 cultures, mitochondrial biogenesis was unable to compensate for increased diabetes-induced mitophagy. This lack of compensation is most likely explained by metabolic (rather than hyperosmotic) stress, since LG cultures counteracted mitochondrial degradation through upregulation of biogenesis. Since the biogenesis machinery appeared unaltered in 2-month-old hyperglycemic *Ins2^Akita/+^* mice, it remains unclear why mitophagy is not appropriately counteracted at the early stages of diabetes. Previous investigations have suggested the dysregulation of mitochondrial biogenesis in the diabetic retina may result, at least in part, to damage to mtDNA replication systems (39); however, this hallmark was observed in *Ins2^Akita/+^* mice only at the advanced stages of the disease. The dysregulation of mitochondrial biogenesis in the hyperglycemic context may have important pathophysiological consequences, including bioenergetic deficits due a net-loss of mitochondrial mass. On the other hand, the reduction of mitochondrial mass may reflect an attempt to decrease the overproduction of mitochondrial ROS in the diabetic retina and/or minimize pathophysiological effects associated with the accumulation of damaged mitochondria, such as cGAS-STING–mediated proinflammatory insult and activation of intrinsic mitochondrial apoptotic pathways (40).

A reduction in the rate of mitophagy was observed at the outer retina in advanced stages of diabetes. The disruption of MQC through the impairment of mitophagy has emerged as major cause of CNS degeneration, since it may lead to the build-up of oxidized mitochondria incompatible with tissue homeostasis (14). Therefore, it is not surprising that impaired mitophagy in *Ins2^Akita/+^* mice was associated with mtDNA damage but also with a disease stage where advanced neurovascular degeneration has been reported (15, 17). Whether the reduction in the rate of mitophagy at advanced stages arises due to failure of autophagy, mitophagy, or both needs further investigation. Previous studies supported the dysregulation of autophagy in the diabetic retina, either suggesting an increase (41) or a deficiency (42) in the flux. Our study concurs with both observations, depending on the duration of diabetes examined. In contrast to 2-month-old hyperglycemic *Ins2^Akita/+^* mice where autophagy appears to be coupled with increased mitophagy flux, at advanced stages of the disease, the accumulation of Lc3b^+^ autophagosomes and p62/SQTSM1 may indicate ineffective autophagy (21). This could arise due to a deficit of cargo degradation in lysosomes, since the levels of mitochondria entering autophagosomes were increased.

We further propose that premature senescence of the outer retina may play an important role in the disruption of MQC, which may shift mitochondrial contents to higher levels at advanced stages of DR. In agreement with this, the accumulation of dysfunctional mitochondria is a well-known hallmark of senescent cells (30). At present, the mechanisms initiating cellular senescence in the diabetic retina remain uncertain. The increase of autophagic flux from the early stages could potentially facilitate the process of senescence (43). Moreover, the dysregulation of MQC due to inefficient mitophagy may also contribute to this process (30). Aside from these factors, other different stressors such as genotoxic or oxidative insult could be relevant (44). Regardless of its origin, the senescence of the diabetic retina may have important pathological implications, such as the development of senescence-associated secretory phenotype (SASP), associated with an exacerbated secretion of proinflammatory mediators (45).

In summary, our study provides insights into the pathobiology of DR, which may be therapeutically relevant for the early and advanced stages of the disease. Therapies aimed at counteracting increased diabetes-induced mitophagy through stimulating mitochondrial biogenesis may be important during the early stages. However, this strategy could involve a risk when the efficiency of mitophagy decreases (i.e., at more advanced stages), which may worsen the accumulation of damaged mitochondria in the diabetic retina. While promoting mitophagy at these stages appears reasonable, future studies will determine the suitability of those therapies for the management of DR.

## Methods

### Animals

Male heterozygous *Ins2^Akita/+^* mice of C57BL/6J background (originally purchased from the Jackson Laboratory) and age-matched ND siblings (WT) were used in the study. The *Ins2^Akita/+^* mice develops severe hyperglycemia (above 550 mg/dL or 30.5 mM) by 4 weeks of age (46). *mitoQC^+/–^ Ins2^Akita/+^* mice were generated by mating *mitoQC^+/+^* females (provided by Ian G. Ganley, University of Dundee, Dundee, United Kingdom) with *Ins2^Akita/+^* males. The diabetic phenotype in the resultant male offspring was corroborated by the levels of glucose (above 550 mg/dL or 30.5 mM) and HbA1c (diabetic *mitoQC^+/–^ Ins2^Akita/+^* had 113.5 ± 4.6 mmol/mol; ND *mitoQC^+/–^ Ins2^+/+^* siblings had 29.7 ± 1.9 mmol/mol). Detection of the *mitoQC*-knockin allele (*mCherry-GFP-mtFIS1^101–153^*) was determined by PCR (47).

### Real-time PCR

Total RNA was isolated from *Ins2^Akita/+^* and age-matched WT retinas (*n* = 6–8 retinas/group) using the RNeasy Mini Kit (Qiagen) and real-time PCR performed using SYBR-Green Master in a Light-Cycler 480 system (Roche Diagnostics). The relative expression of target genes (Supplemental Table 1) was normalized to *18s*.

### Mitochondrial copy number

Total DNA from *Ins2^Akita/+^* and age-matched WT retinas (*n* = 7–10 retinas/group) was extracted using the DNeasy Blood & Tissue Kit (Qiagen), and real-time PCR was performed using specific primers (Supplemental Table 1) to detect *mMITO* and *cytochrome-c oxidase subunit II* (*CoII*) as markers for mtDNA and *18s* for nuclear DNA (nDNA). The mtDNA/nDNA ratio was used as measurement of mtDNA copy number.

### mtDNA damage

Total DNA from *Ins2^Akita/+^* and age-matched WT retinas (*n* = 7–10 retinas/group) was amplified by PCR using specific primers (Supplemental Table 1) for long (10.1 Kb) and short (116 pb) mtDNA regions (48). Long and short amplification products were respectively separated in 1% and 2% agarose gels, and the intensity of SYBR_Safe DNA blots was quantified using FIJI software (NIH). The relative amplification of the long PCR product was normalized to the short product; a reduction in the amplification ratio was indicative of increased mtDNA damage (48).

### Intravitreal injection of chloroquine

Autophagy flux in the retina was blocked via intravitreal administration of 1 μL chloroquine (Thermo Fisher Scientific; 500 μM) in 3-month-old WT mice. Injections were performed as previously reported (49). Twenty-four hours following chloroquine administration, mouse eyes were collected and processed for IHC.

### IHC

#### Human retinas.

Age-matched human retinas from diabetic and ND individuals were obtained postmortem from the National Disease Research Interchange (Philadelphia, Pennsylvania, USA) as described (41). The groups were categorized as ND (*n* = 3 donors), DNR (*n* = 5 donors; type-1 diabetes [*n* = 3], type-2 diabetes [*n* = 2]), and diabetes with retinopathy (*n* = 2 donors; type-2 diabetes, nonproliferative DR). Following deparaffinization, retinal sections were immersed (1 hour) in antigen retrieval buffer (EDTA, pH 8.0; Thermo Fisher Scientific) at 60°C. Sections were then rinsed in PBS and incubated overnight (4°C) with Cox4 and cone arrestin antibodies (Supplemental Table 2) as previously described (50).

#### Mouse retinas.

Eyes were dissected, fixed in 2% paraformaldehyde (Sigma-Aldrich), and processed for IHC (Supplemental Table 2) as previously described (50).

### Cell culture

The human Müller cell line MIO-M1 was obtained from the UCL Institute of Ophthalmology (London, United Kingdom; ref. 51). PMCs from *mitoQC^+/+^* mice were isolated and cultured as previously described (25). PMCs were used for experiments from P2–P6, where most cells showed a senescence phenotype. Cultures were maintained in DMEM (containing 10% FCS [Thermo Fisher Scientific], 100 U/mL penicillin-streptomycin [Sigma-Aldrich]) and supplemented with 5.5 mM D-glucose (NG), 30.5 mM D-glucose (Sigma-Aldrich; HG), or 30.5 mM LG (Alfa aesar; 25 mM LG + 5.5 mM NG) for 5 days. The selection of 30.5 mM D-glucose was based on the levels of hyperglycemia found in the plasma of *Ins2^Akita/+^* mice. For mitophagy-induced amino acid starvation, cultures were maintained in HBSS (Thermo Fisher Scientific; 16 hours). Autophagy flux was blocked with 100 μM chloroquine (12 hours). Endpoint experiments were performed in 70%–80% confluent cultures. No mycoplasma was detected in the cell cultures (PCR Mycoplasma Test Kit I/C; PromoCell).

### Immunocytochemistry

Cells were fixed in 2% paraformaldehyde, rinsed in PBS, and blocked (3% BSA [Sigma-Aldrich], 0.1% TritonX-100 [Sigma-Aldrich], PBS). Cells were then incubated overnight (4°C) with primary antibodies (Supplemental Table 2) diluted in 3% BSA and 0.05% Tween-20 [Sigma-Aldrich], PBS. Following incubation, cells were probed (1 hours) with fluorophore-conjugated secondary antibodies (all from Jackson ImmunoResearch) at room temperature.

### Western blotting

Retinas and MIO-M1 cells were lysed in RIPA buffer with protease and phosphatase inhibitors cocktails (Sigma-Aldrich). Protein samples (10–20 μg) were run on 7.5%, 10%, or 12% (w/v) SDS-PAGE gel, and samples were immunoblotted for primary antibodies (Supplemental Table 2). Immunoblots (obtained from 3 biological replicates) were quantified by densitometry, and protein expressions were normalized to β-actin or α-tubulin levels.

### pMitoTimer transfections

*pMitoTimer* (Addgene) incorporates a fluorescent timer reporter to mitochondria (*pDsRed2-Mito*) that fluoresces GFP when newly synthetized and irreversibly shifts to red spectrum (excitation/emission [Ex/Em] 558/583 nm) over time (28). MIO-M1 cells were incubated (12 hours) with a mixture of 50 ng plasmid DNA and 0.15 μL endofectin (GeneCopoeia) in Opti-MEM (Thermo Fisher Scientific). Cells were then maintained in NG, HG, or LG and fixed for microscopy.

### JC-1 dye staining

The ψm was assessed by ratiometric analysis of JC-1 (Thermo Fisher Scientific). Following NG, HG, or LG treatment, MIO-M1 cultures were supplemented with 0.5 μg/mL JC-1 (30 minutes at 37°C) and returned to DMEM for microscopy. Positive controls were supplemented for 16 hours with carbonyl cyanide m-chlorophenylhydrazone (CCCP, 20 μM; Abcam) to uncouple mitochondria.

### Immunolabeling BrDU DNA

Following NG, HG, or LG treatment, cultures were supplemented (12 hours) with 10 μM BrdU (Thermo Fisher Scientific) and fixed. Cells were then rinsed in PBS and the DNA denatured (20 min) with 0.5 M HCl. Cells were washed with PBS and processed for anti-BrDU immunocytochemistry.

### SA-β-Gal activity

Retinal cryosections obtained from *Ins2^Akita/+^* and WT mice (*n* = 6 eyes/group) and *mitoQC*-PMCs cultures (P2–P3 and P5–P6) were processed following manufacturer’s instructions of the Senescence Detection Kit (Abcam).

### Confocal morphometry

Confocal images were acquired under constant photomultiplier settings (C1-Nikon_Eclipse TE200-U) and analyzed using FIJI software. To avoid any bias during imaging, retinal regions were selected based on the DAPI nuclear signal and invariably, from middle-center eccentricities. For cell cultures, images were selected from the same cardinal points of the wells using bright-field imaging.

#### Cox4 levels in human retinal sections.

Images (2 retinal sections/eye; 8 images/section) were used to quantify the mean fluorescence intensity (MFI) of Cox4. For this purpose, mean luminance values (average brightness per pixel) were calculated from manually traced areas, including (a) IS of cone photoreceptors (identified by cone arrestin immunoreactivity), (b) OPL, and (c) IPL. Background was acquired from a vacant area of the labeled section and subtracted from the raw images to eliminate background noise. The technical replicates (*n* = 16 for each eye) were used for statistical analysis.

#### MFI in mouse retinas.

Images (*n* = 4–5 eyes/group; 2 retinal sections/eye; 4 images/section) were used to quantify the MFI values of Cox4, PGC-1α, Lc3b, and p62/SQTSM1 in WT and *Ins2^Akita/+^* mice. Measurements were obtained (a) from the photoreceptor IS to the OPL (referred as the outer retina) and (b) from the INL to the GCL (referred as the inner retina). MFI values were averaged for each eye.

#### Ubiquitin in mitochondria of photoreceptor IS.

Images (*n* = 5–6 eyes/group; 2 retinal sections/eye; 4 images/section) were used to delineate mitochondrial ROIs (Fis1^+^ area) in photoreceptor IS. The MFI of ubiquitin immunostaining was then assessed in mitochondrial ROIs. MFI values were averaged for each eye.

#### Quantification of TFAM^+^mitochondrial nucleoids and TOMM20^+^ mitochondria at the outer retina.

Mitochondrial nucleoids and TOMM20^+^ mitochondria were quantified in confocal retinal images (*n* = 5–8 eyes/group; 2 retinal sections/eye; 4 images/section) by threshold image binarization of TFAM^+^ or TOMM20^+^ particles at the IS-OPL (constant values were applied for all groups), and their number was obtained by particle analysis in FIJI. Mitochondrial nucleoid and TOMM20^+^ mitochondrial values were then normalized to the outer retinal area analyzed. Values were averaged for each eye.

#### Quantification of mitolysosomes (mCherry-only foci) at the outer retina.

The total mitolysosome number in confocal retinal images (*n* = 4–8 eyes/group, 2 retinal sections/eye; 4 images/section) at the outer retina (IS-OPL) was determined by the subtraction of GFP signal from mCherry using the “image calculator” plugin of FIJI. This was followed by threshold image binarization of mitolysosomes (constant values were applied for all groups), and the total number was obtained by particle analysis. Mitolysosome number was then normalized to the outer retinal area analyzed. Values were averaged for each eye.

#### SA-β-Gal in mouse retinas.

Images (*n* = 6 eyes/group; 2 retinal sections/eye; 4 images/section) were inverted and transformed into 32-bit color to quantify the intensity of SA-β-Gal staining in WT and *Ins2^Akita/+^* mice. Measurements obtained from the IS were averaged for each eye.

#### Quantification of mitolysosomes in mitoQC-PMCs.

The total mitolysosome area in individual cells was determined by the subtraction of GFP signal from mCherry using the “image calculator” plugin of FIJI. This was followed by threshold image binarization of mitolysosomes (constant values were applied for all groups), and the total area (μm^2^) was obtained by particle analysis. Values (P2–P3 cells, *n* = 3–5 biological replicates per group; P5–P6 cells, *n* = 2 biological replicates and 4 technical replicates per group) were normalized to the cellular area.

#### BrDU incorporation in mtDNA.

The cytoplasmic area of each individual cell was manually traced, inverted, and duplicated for analysis. The BrDU^+^ area was then delineated by threshold image binarization (using constant values for all groups), and the total area (μm^2^) was obtained by particle analysis. Values were normalized to the cellular area. At least 70 cells (obtained from *n* = 3 biological replicates) were analyzed. The specificity of BrDU within mitochondria was validated by costaining with TOMM20 (data not shown).

#### Ratiometric analysis of pMitoTimer.

Red- and GFP-fluorescent signals were merged and *pMitoTimer* ROIs obtained by threshold image binarization and particle analysis. Red- and GFP-fluorescent MFIs were then obtained from the *pMitoTimer* ROIs and the R/G ratio was calculated in each image. Cells obtained from *n* = 2 biological replicates (5 technical replicates per group) were analyzed.

#### Colocalization analysis in retina.

Images (*n* = 5 eyes/group; 2 retinal sections/eye; 2 images/section) were processed for colocalization analysis using the Intensity Correlation Analysis of the WCIF-ImageJ module (52). The total colocalizing area (μm^2^) was then obtained by threshold image binarization and particle analysis of the generated +ves stack (which shows all positive colocalizing pixels). The percentage of Cox4 or Pink1, respectively colocalized with Lc3b or LAMP1, was determined as = (total colocalizing area) × 100/(Cox4 [or] Pink1 area). For each eye, values were averaged. Pink1 antibody was validated using *MitoQC^+/+^Pink1^–/–^* mouse eyes (provided by Ian G. Ganley) (Supplemental Figure 10A). Pink1, Lc3b, and LAMP1 antibodies were further validated for IHC via intravitreal injection of chloroquine in mouse eyes (Supplemental Figure 10, B–D).

#### Colocalization analysis in MIO-M1 cells.

The total colocalizing area between Cox4 and Lc3b (calculated as above) was normalized to the cell area. Data were then referred as fold changes to NG. At least 70 cells (obtained from *n* = 3 biological replicates) were analyzed.

### Statistics

In each age group, the difference between 2 means was analyzed using 2-sided unpaired Student’s *t* test and 1-way ANOVA (followed by Bonferroni’s post hoc analysis) used for comparisons with more than 2 groups (GraphPad Prism). To compare the difference between 2 means by immunohistochemical morphometry, sample size was adjusted to *n* = 4–9 eyes in each mouse group based on an 80% power and a 5% significant level. Significant out-layers were discarded using Grubbs’ test (α = 0.05). Data were expressed as mean ± SEM. *P* < 0.05 was considered statistically significant.

### Study approval

The study was approved by the Ethics Committee at the Queen’s University of Belfast and IRB at the University of Oklahoma Health Sciences Centre (OUHSC). Human studies were conducted according to the Declaration of Helsinki principles, and written informed consent was received from participants prior to inclusion in the study. All animal procedures were approved by Ethical Review Body (AWERB) and authorized under the UK Animals (Scientific Procedures) Act 1986. Animal use conformed to the standards in the Association for Research in Vision and Ophthalmology (ARVO) Statement for the Use of Animals in Ophthalmic and Vision Research and with European Directive 210/63/EU.

## Author contributions

JRH conceived and designed the experiments with input from TMC, PM, and HX; JRH, LC, and LRD performed the experiments; JRH and LC analyzed the data; TJL contributed with the human retinal samples; DPB and HX contributed with reagents/materials; JRH and TMC wrote and edited the manuscript with input from all other authors. JRH supervised the project.

## Supplementary Material

Supplemental data

## Version 1 10/29/2019

In-Press Preview

## Version 2 12/05/2019

Electronic publication
