# Supplementary material for: Uncoupled turnover disrupts mitochondrial quality control in diabetic retinopathy
Source: JCI Insight. 2019 Dec 5;4(23):e129760. doi: 10.1172/jci.insight.129760 (PMC6962019; doi:10.1172/jci.insight.129760)
Supplement: Supplemental data [file jciinsight-4-129760-s009.pdf]

Uncoupled turnover disrupts mitochondrial quality control in diabetic retinopathy

Jose R. Hombrebueno[1,2#], Lauren Cairns[1], Louise R. Dutton[1], Timothy J. Lyons[1,3], Derek P. Brazil[1], Paul Moynagh[1,4], Tim M. Curtis[1], Heping Xu[1]

[1]Wellcome-Wolfson Institute for Experimental Medicine, School of Medicine, Dentistry and Biomedical Sciences, Queen's University Belfast, Belfast, UK.

[2]Institute of Inflammation and Ageing, University of Birmingham, Birmingham, UK

[3]Division of Endocrinology and Diabetes, Medical University of South Carolina, Charleston, SC, USA.

[4]Institute of Immunology, Department of Biology, National University of Ireland Maynooth, Maynooth, County Kildare, Ireland

[#]Correspondence to Dr Jose Romero Hombrebueno; j.m.romero@bham.ac.uk

Institute of Inflammation and Ageing, School of Medical and Dental Sciences, University of Birmingham, Edgbaston, Birmingham, B15 2TT, UK. Phone: +44 1213713226

The authors have declared that no conflict of interest exists

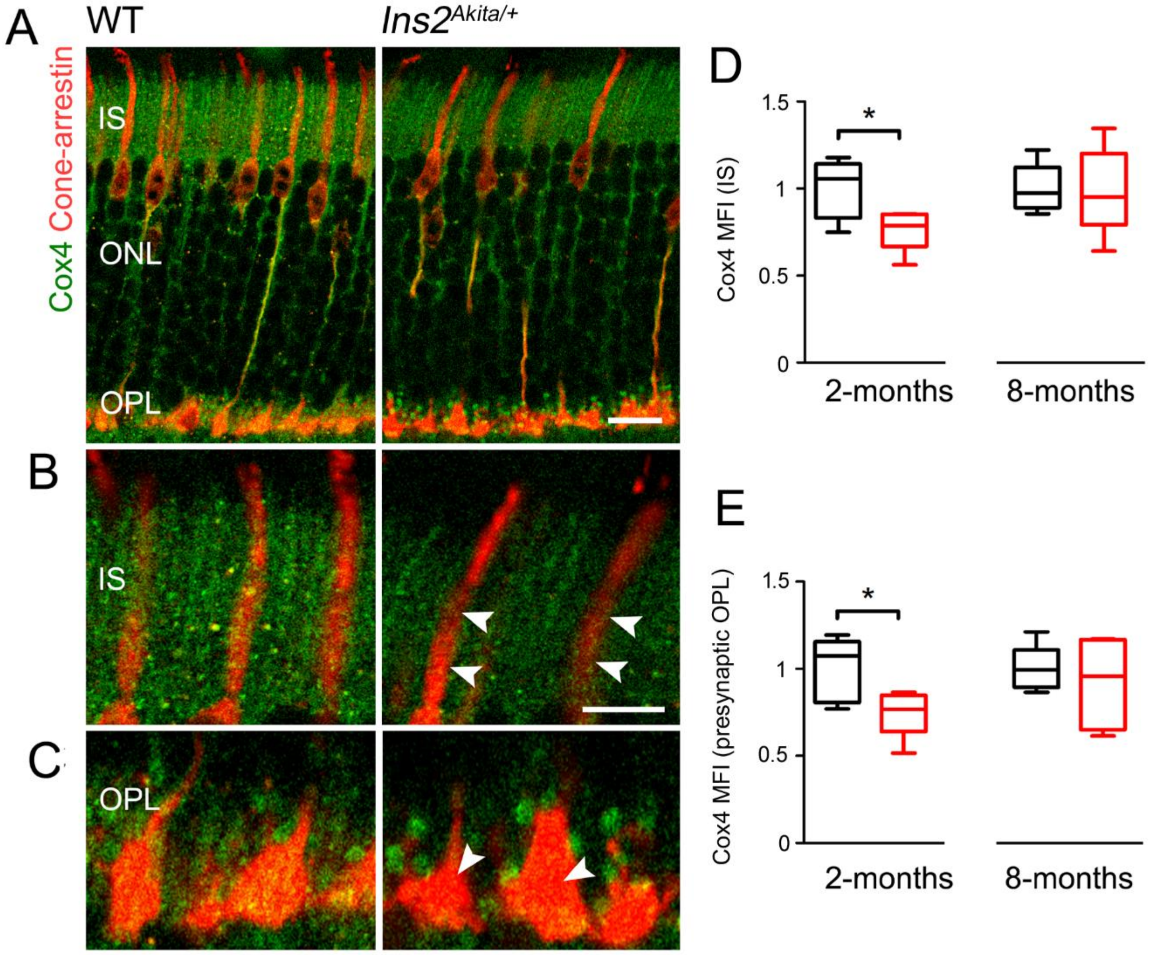

Supplemental Fig 1. Photoreceptor mitochondrial contents change during the progression of diabetes in *Ins2$^{Akita/+}$* retinas. (A-C) Retinal micrographs of 2-month hyperglycaemic *Ins2$^{Akita/+}$* and age-matched WT mice processed for Cox4 and cone-arrestin immunostaining. Arrowheads indicate loss of Cox4 within cone photoreceptors IS (B) and synaptic terminals of the OPL (C). (D-E) The mean fluorescence intensities (MFI) of Cox4 at the IS (D) and OPL (E) of 2-month and 8-month hyperglycaemic *Ins2$^{Akita/+}$* and age-matched WT mice. Keys: WT (black plots), *Ins2$^{Akita/+}$* (red plots). Data is presented in box-and-whisker plots; n=5 eyes per strain. Results presented as mean ± SEM. *$p<0.05$, two-sided unpaired Student's t-test. IS, photoreceptor inner segments; OPL, outer plexiform layer; ONL, outer nuclear layer. Scale bar = 20 μm (A), 10μm (B).

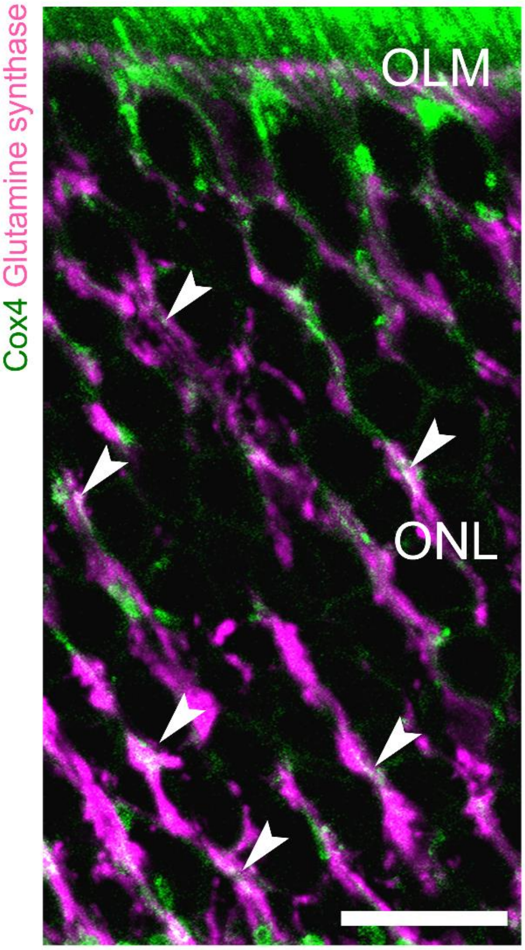

Supplemental Fig 2. Müller cell processes contain high levels of mitochondria at the outer retina. Retinal micrographs of 2-month WT mice processed for Cox4 and glutamine synthase (GS) immunostaining. The arrowheads indicate enrichment of $Cox4^+$ mitochondria within GS^+ Müller cell processes at the outer nuclear layer (ONL). OLM, outer limiting membrane. Scale bar = 20 µm

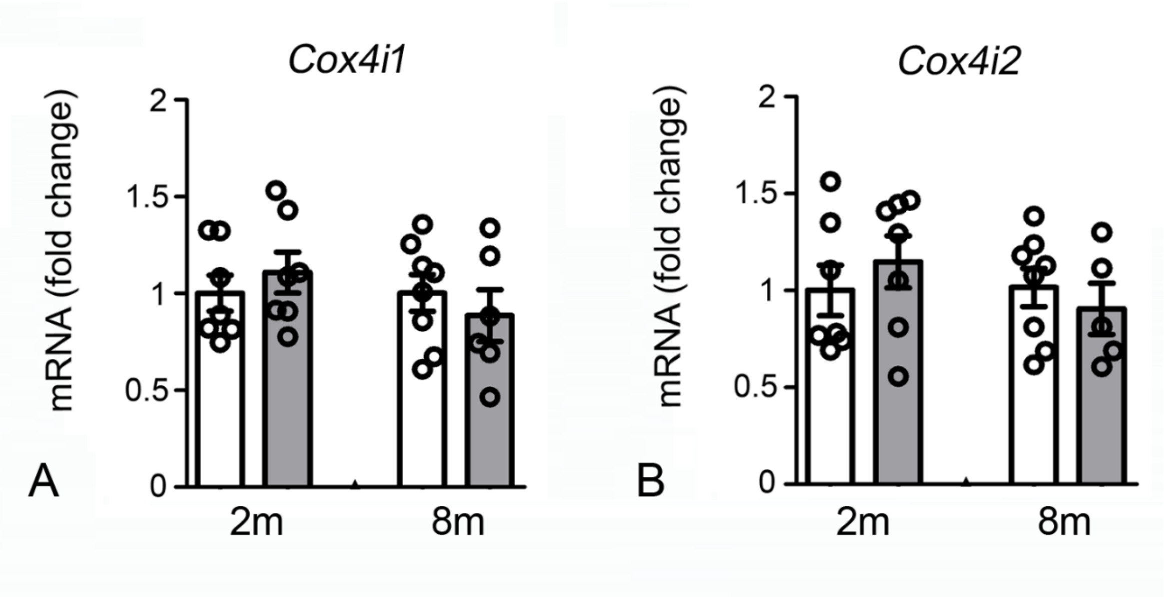

Supplemental Fig 3. Cox4 mRNA levels in 2-month and 8-month hyperglycaemic *Ins2$^{Akita/+}$* retinas. rtPCR analysis of *Cox4i1* (A) and *Cox4i2* (B) isoforms in the retina of 2-month and 8-month hyperglycaemic *Ins2$^{Akita/+}$* and age-matched WT mice. Keys: WT (white bars), *Ins2$^{Akita/+}$* (grey bars); n=5-8 eyes per strain. Results are presented as mean ± SEM. Two-sided unpaired Student's *t*-test.

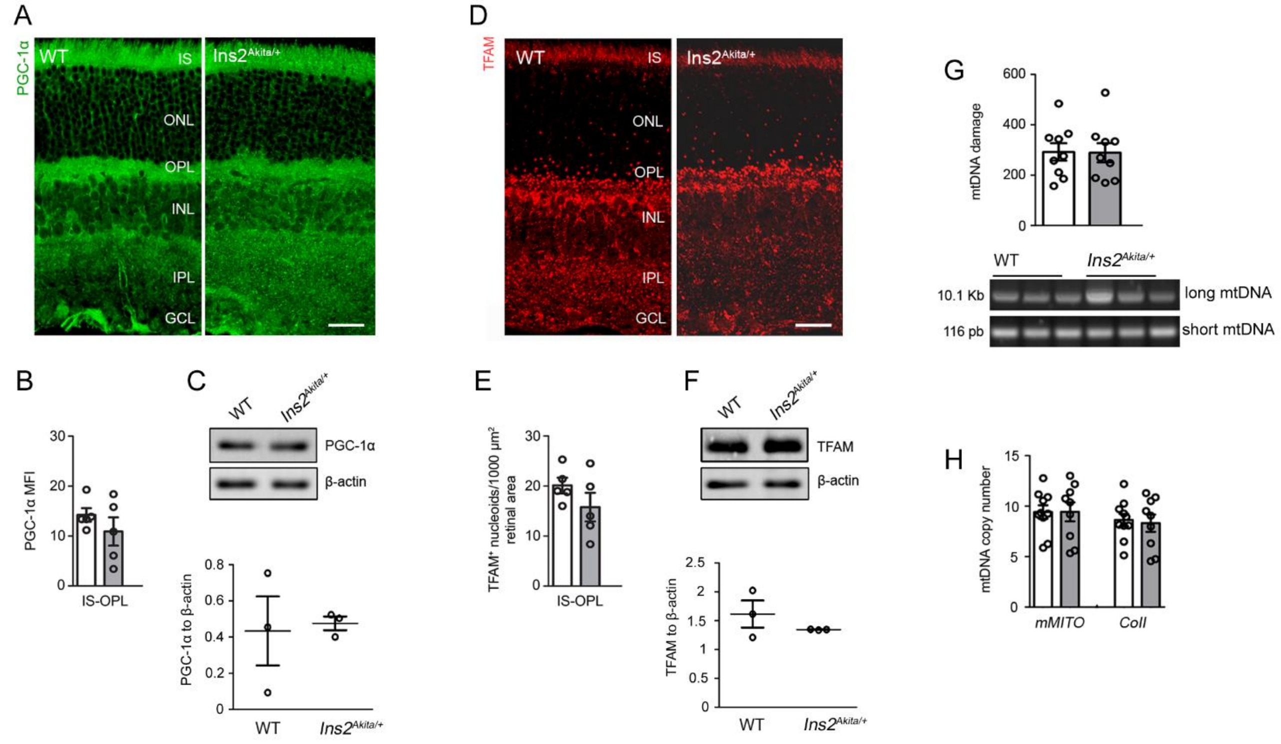

Supplemental Fig 4. Mitochondrial biogenesis machinery in 2-month hyperglycaemic *Ins2*$^{Akita/+}$ mouse retinas. (A) Retinal micrographs from 2-month hyperglycaemic *Ins2*$^{Akita/+}$ and aged-matched WT mice processed for PGC-1α immunostaining. (B) The mean fluorescence intensity (MFI) of PGC-1α at the IS-OPL. (C) Example immunoblot and quantification of PGC-1α in mouse retinal lysates of 2-month hyperglycaemic *Ins2*$^{Akita/+}$ and age-matched WT. Data was normalized to β-actin loading controls. (D) Retinal micrographs from 2-month hyperglycaemic *Ins2*$^{Akita/+}$ and aged-matched WT mice processed for TFAM immunostaining. (E) The density of TFAM^{+} mitochondrial nucleoids at the IS-OPL. (F) Example immunoblot and quantification of TFAM in mouse retinal lysates of 2-month hyperglycaemic *Ins2*$^{Akita/+}$ and age-matched WT. Data was normalized to β-actin loading controls. TFAM shared similar β-actin loading controls to those in Fig 2A (Cox4) (G) Evaluation of mtDNA damage in 2-month hyperglycaemic *Ins2*$^{Akita/+}$ and age-matched WT mouse retinas by amplification of long (10.1Kb) and short (116pb) mtDNA regions. A reduction in the long/short amplification ratio is indicative of mtDNA damage. (H) Mitochondrial copy numbers evaluated by qPCR analysis of *mMITO* and *CoII* mtDNA regions. Keys: WT (white bars), *Ins2*$^{Akita/+}$ (grey bars); n=3-10 eyes per strain. Results are presented as mean ± SEM. Two-sided unpaired Student's *t*-test. IS, photoreceptor inner segments; ONL, outer nuclear layer; OPL, outer plexiform layer; INL, inner nuclear layer; IPL, inner plexiform layer; GCL, ganglion cell layer. Scale bars = 40 µm.

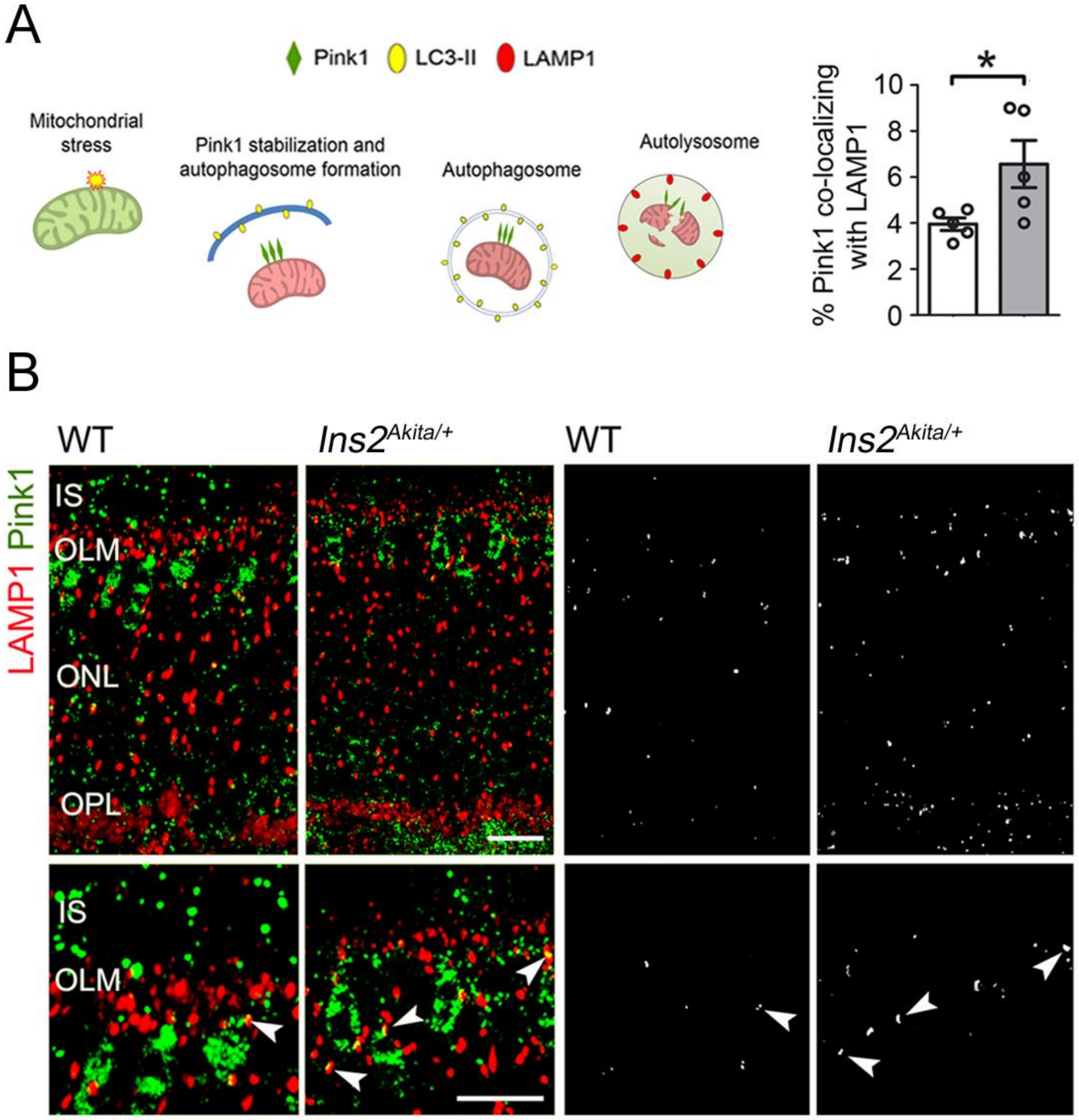

Supplemental Fig 5. Elevated Pink1-mitophagy at the outer retina of 2-month hyperglycaemic *Ins2$^{Akita/+}$* mice. (A) The levels of Pink1 within lysosomes were assessed as an index of Pink1-dependent mitophagy in the retina of 2-month hyperglycaemic *Ins2$^{Akita/+}$* and age-matched WT mice. (B) Confocal photomicrographs show Pink1-LAMP1 co-localizing particles (as highlighted by particle binarization) at the IS-OPL (arrowheads). Keys: WT (white bars), *Ins2$^{Akita/+}$* (grey bars), n=5 eyes per strain. Results presented as mean ± SEM. *p<0.05, two-sided unpaired Student's t-test. IS, photoreceptor inner segments; OLM, outer limiting membrane, ONL, outer nuclear layer; OPL, Scale bars = 20µm.

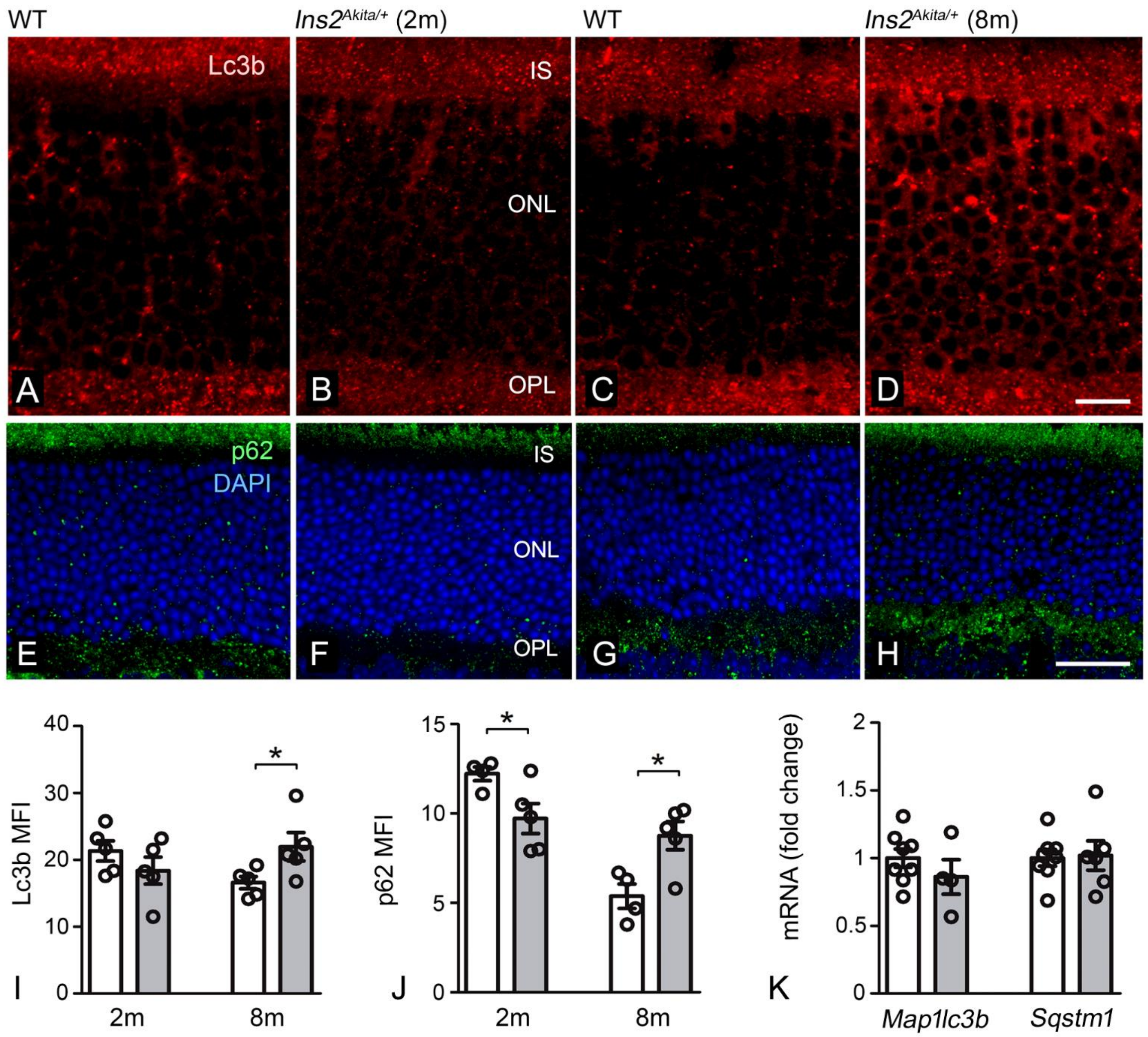

Supplemental Fig 6. Autophagy adaptors accumulate during the progression of diabetes in *Ins2$^{Akita/+}$* retinas. (A-H) Retinal micrographs of 2 and 8-month hyperglycaemic *Ins2$^{Akita/+}$* and aged-matched WT mice processed for Lc3b (A-D) and p62/SQTSM1 (E-H) immunoreactivities. The mean fluorescence intensities (MFI) of Lc3b (I) and p62/SQTSM1 (J) at the outer retina (IS-OPL) of 2-month and 8-month hyperglycaemic *Ins2$^{Akita/+}$* and aged-matched WT mice. rtPCR analysis of *Map1lc3b* and *Sqtsm1* gene transcripts in the retina of 8-month hyperglycaemic *Ins2$^{Akita/+}$* and age-matched WT mice. Keys (I-K): WT (white bars), *Ins2$^{Akita/+}$* (grey bars), n=4-8 eyes per strain. Results are presented as mean ± SEM. *$p < 0.05$. Two-sided unpaired Student's *t*-test. IS, photoreceptor inner segments; ONL, outer nuclear layer; OPL, outer plexiform layer. Scale bars = 10 µm (D), 40 µm (H).

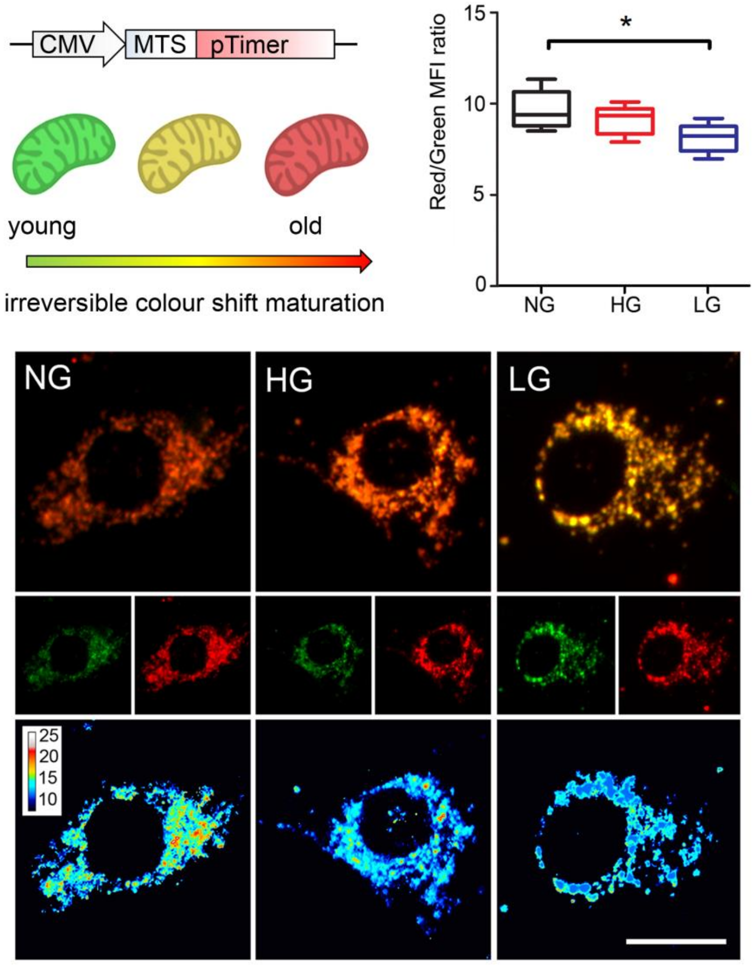

Supplemental Fig 7. Evaluation of mitochondrial turnover in MIO-M1 cultures with *pMitoTimer*. MIO-M1 cells were transfected with *pMitoTimer* and then maintained for 5 days in normal glucose (NG - 5.5mM), high glucose (HG - 30.5mM) or L-glucose (LG – 30.5mM) osmotic control. The lower images show the red/green ratio in false colour, with a low red-to-green ratio (indicative of younger mitochondria) in blue. Quantification of *pMitoTimer* red-to-green ratio is presented in box-and-whisker plots; n=2 biological replicates and 5 technical replicates per group. One-way ANOVA with Bonferroni's correction for multiple comparisons. CMV, cytomegalovirus promoter; MTS, mitochondrial targeting sequence; MFI, mean fluorescence intensity. Scale bar= 20 µm.

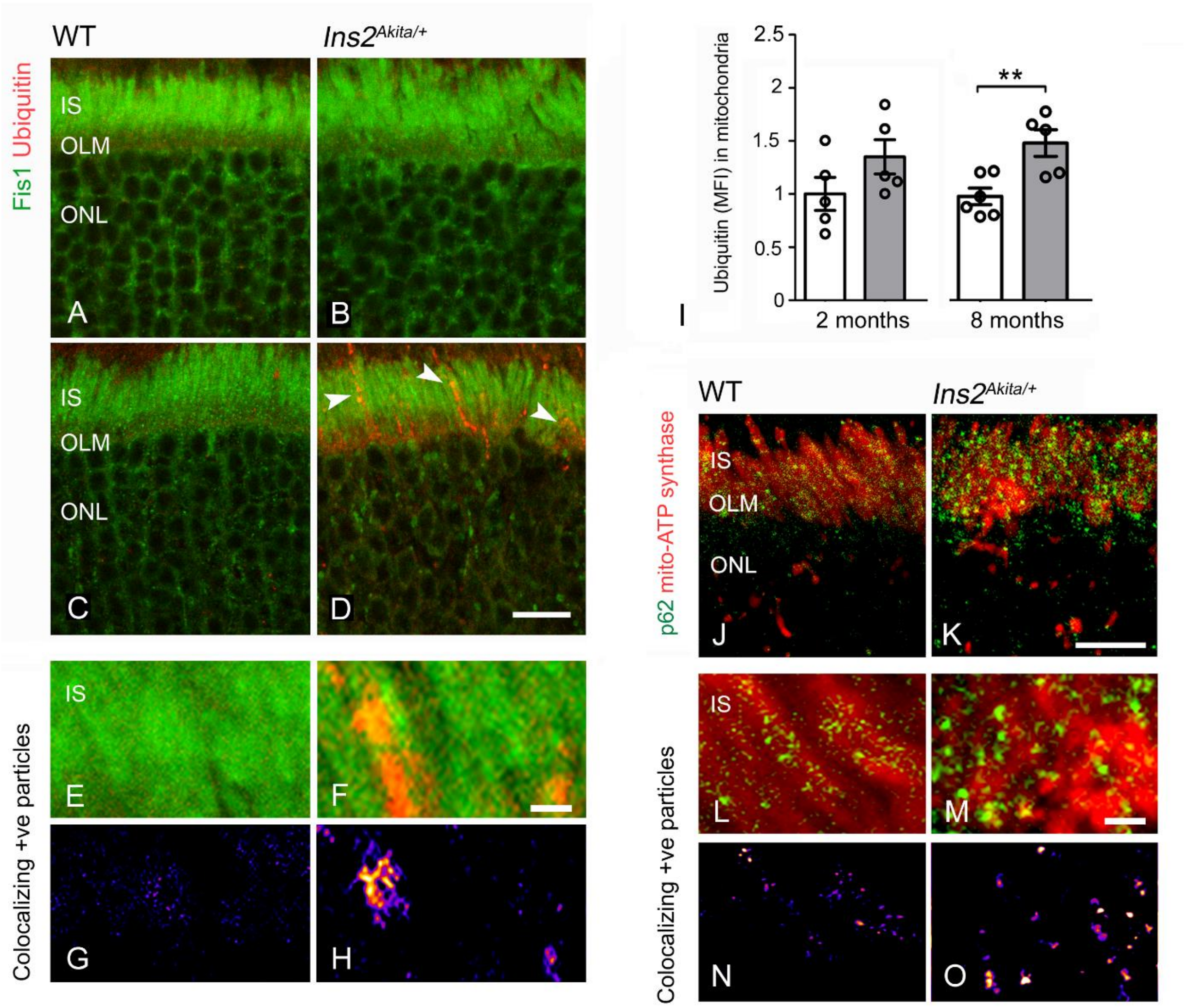

Supplemental Fig 8. Accumulation of ubiquitin and p62 in mitochondria at the outer retina of 8-month hyperglycaemic *Ins2*$^{Akita/+}$ mice. Retinal micrographs of 2-month (A-B) and 8-month (C-H) hyperglycaemic *Ins2*$^{Akita/+}$ and aged-matched WT mice processed for Fis1 and Ubiquitin immunoreactivities. Arrowheads in (D) indicate ubiquitin accumulation in mitochondria of photoreceptor IS. (E-F) High magnification images of Fis1 and Ubiquitin immunoreactivities at the inner segments (IS) of photoreceptors. (G-H) Fis1-Ubiquitin co-localization is shown in false colour (higher values: white-orange, low values: purple-black). (I) The mean fluorescence intensities (MFI) of ubiquitin in the mitochondria of photoreceptor IS of 2-month and 8-month hyperglycaemic *Ins2*$^{Akita/+}$ and aged-matched WT mice. (J-K) Retinal micrographs of 8-month hyperglycaemic *Ins2*$^{Akita/+}$ and aged-matched WT mice processed for p62 and mitochondrial ATP-synthase immunoreactivities. (L-M) High magnification insets of p62 and mitochondrial ATP-synthase in photoreceptor IS. (N-O) p62 co-localization with mitochondrial ATP-synthase is shown in false colour (higher values: white-orange, low values: purple-black). Keys: WT (white bars), *Ins2*$^{Akita/+}$ (grey bars); n=5-6 eyes per strain. Results are presented as mean ± SEM (as fold changes compared to age-matched WT mice). $^{**}p < 0.01$. Two-sided unpaired Student's *t*-test. OLM, outer limiting membrane; ONL, outer nuclear layer. Scale bars = 10 µm (D, K), 2 µm (F, M).

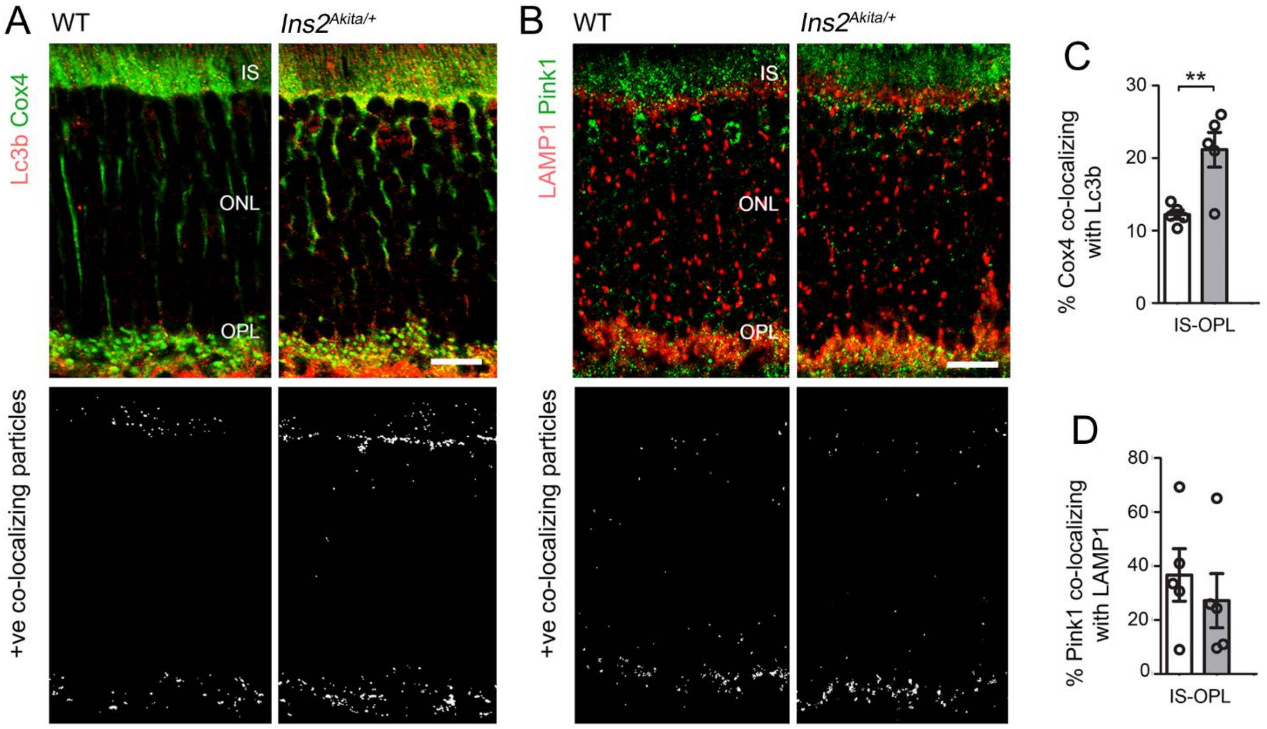

Supplemental Fig 9. Inefficient mitophagy at the outer retina of 8-month hyperglycaemic *Ins2*$^{Akita/+}$ mice. Representative micrographs and quantification analysis of Lc3b-Cox4 (A, C) and LAMP1-Pink1 (B, D) co-localizing particles at the outer retina (IS-OPL) of 8-month hyperglycaemic *Ins2*$^{Akita/+}$ and aged-matched WT mice. Keys: WT (white bars), *Ins2*$^{Akita/+}$ (grey bars); n=5 eyes per strain, Results are presented as mean ± SEM. **p<0.01, two-sided unpaired Student's t-test. IS, photoreceptor inner segments; ONL, outer nuclear layer; OPL, outer plexiform layer. Scale bars = 20 μm (A-B).

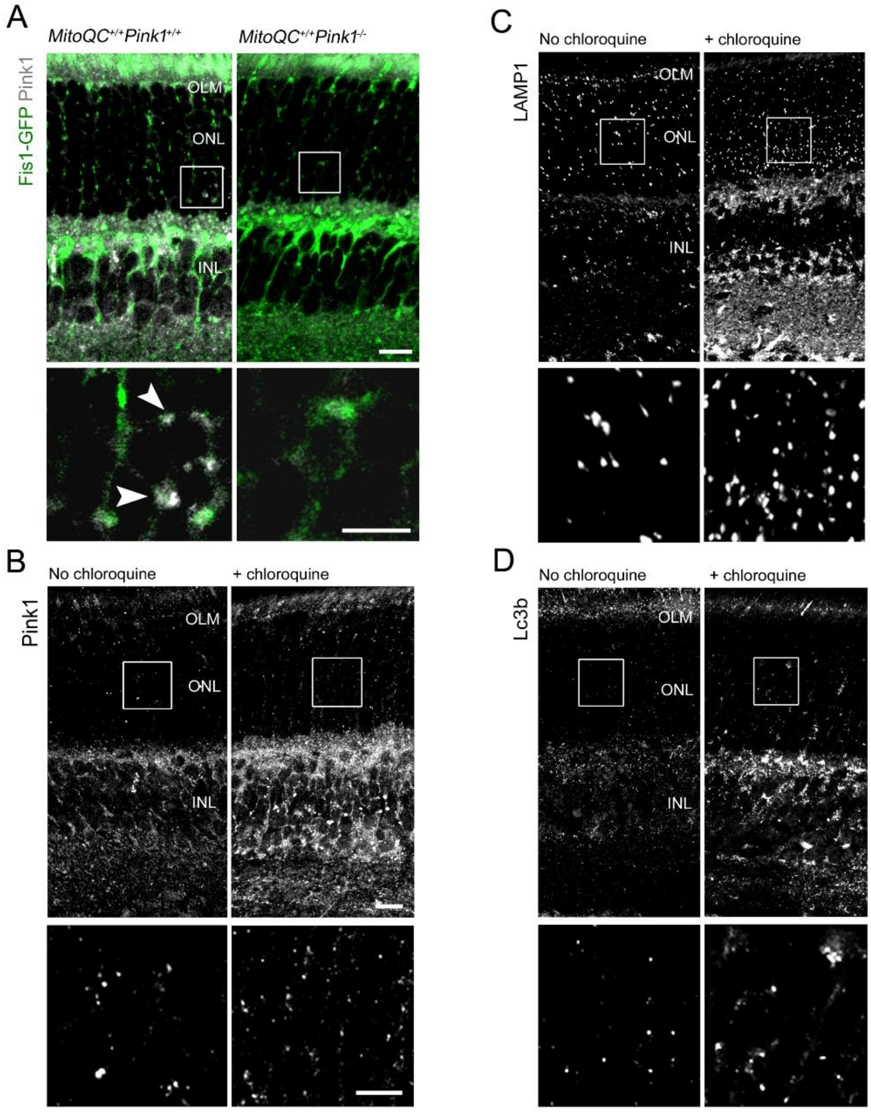

Supplemental Fig 10. Validation of Pink1, Lc3b and LAMP1 antibodies for immunohistochemical analysis in mouse retinas. Representative confocal photomicrographs of Pink1 (A, B), LAMP1 (C) and Lc3b (D) immunoreactivities in the retina of (A) *MitoQC*$^{+/+}$*Pink1*$^{+/+}$ and *MitoQC*$^{+/+}$*Pink1*$^{-/-}$ mice or (B-D) C57BL6/J mice 24h after intravitreal injection of 500 µM chloroquine. Enclosed areas indicate high magnification insets (shown below their corresponding panel). (Inset in A) Arrowheads indicate localization of Pink1 immunoreactivity in Fis1-GFP^{+} mitochondria. Insets in (B-D) show accumulation of Pink1 (B), LAMP1^{+} lysosomes (C) and Lc3b^{+} autophagosomes (D) in chloroquine injected eyes as compared to non-chloroquine treated controls. OLM, outer limiting membrane; ONL, outer nuclear layer; INL, inner nuclear layer. Scale bars = 20 µm (A, B), 10 µm (insets in A-B).

Supplemental Tables

Supplemental Table 1. Mouse primers used for PCR and rtPCR

Genes	Forward	Reverse
Cox4i1	TCCCCACTTACGCTGATCG	GATGCGGTACAACTGAACTTTCT
Cox4i2	GTTGACTGCTACGCCCAGCGC	CCGGTACAAGGCCACCTTCTC
Pink1	GCTTGCCAATCCCTTCTATG	CTCTCGCTGGAGCAGTGAC
Park2	TCAGGTTCAACTCCAGCTATGGC	TCCCGGCAAAAATCACACGCAGC
Map1lc3b	TTATAGAGCGATACAAGGGGGAG	CGCCGTCTGATTATCTTGATGAG
Sqstm1	GAGGCACCCCGAAACATGG	ACTTATAGCGAGTTCCCACCA
mMITO	CTAGAAACCCCGAAACCAAA	CCAGCTATCACCAAGCTCGT
CoII	ATTGCCCTCCCCTCTCTACGCA	CGTAGCTTCAGTATCATTGGTGCCC
Long mtDNA	GCCAGCCTGACCCATAGCCATAATAT	GAGAGATTTTATGGGTGTAATGCGG
Short mtDNA	CCCAGCTACTACCATCATTCAAGT	GATGGTTTGGGAGATTGGTTGATGT
18s	AGGGGAGAGCGGGTAAGAGA	GGACAGGACTAGGCGGAACA

Supplemental Table 2. Primary antibodies used for western blot (WB), immunohistochemistry (IHC) and immunocytochemistry (IC)

Antigen	Antiserum (Host)	Dilution	Source (catalog number)
ATP Synthase (beta)	mouse	1:150 (IHC)	Thermo (A-21351)
β-actin	mouse	1:10000 (WB)	Santa Cruz (sc-47778)
BrDU	mouse	1:50 (IC)	Sigma (B8434)
Cone-arrestin	rabbit	1:10000 (IHC)	Chemicon (ab15282)
Cox4	goat	1:50 (IHC)	R&D systems (AF5814)
Cox4	mouse	1:200 (IC) - 1:1000 (WB)	Novus Biol (NBP2-59776)
Fis1	rabbit	1:500 (IHC)	Genetex (GTX111010)
Glutamine synthase	rabbit	1:1000 (IC)	Sigma (G2781)
Ki67	rat	1:100 (IC)	Thermo (14-5698-82)
Lc3b	rabbit	1:500 (IHC and IC)	Cell Signalling (3868)
LAMP1 (CD107a)	rat	1:200 (IHC)	BioRad (MCA4707T)
PARL	rabbit	1:1000 (WB)	Abcam (ab45231)
Parkin	mouse	1:1000 (WB)	Abcam (ab77924)
PGC-1α	rabbit	1:5000 (WB) - 1:500 (IHC and IC)	Novus Biol (NBP1-04676)
Pink1	rabbit	1:2000 (WB) - 1:200 (IHC)	Abcam (ab23707)
p62/SQTSM1	rabbit	1:200 (IHC)	Abcam (ab91526)
TFAM	Rabbit	1:1000 (WB) - 1:500 (IHC)	Genetex (GTX112760)
TOMM20	rabbit	1:500 (IHC)	Sigma (HPA011562)
Ubiquitin	mouse	1:1000 (IHC)	Novus Biol (NB300-130)
